# Supplementary material for: Insights into X-Linked Susceptibility to Parkinson’s Disease in the South African Population
Source: medRxiv. 2025 Dec 26:2025.12.23.25342891. Preprint. [Version 1] doi: 10.64898/2025.12.23.25342891 (PMC12755251; doi:10.64898/2025.12.23.25342891)
Supplement: Supplement 1 [file media-1.pdf]

X-Chromosome Wide Association Study of Parkinson's Disease in the South African Population

Kathryn Step, Emily Waldo, Thiago Peixoto Leal, Marla Mendes de Aquino, Soraya Bardien, Ignacio F Mata

Table of contents

Supplementary methods 3

P-value threshold calculations 3

P-value threshold calculations for males 3

P-value threshold calculations for females 3

Supplementary figures 4

Supplementary Figure 1: Principal component analysis for (A) Females PC1 vs PC2, (B) Females PC3 vs PC4, (C) Males PC1 vs PC2, and (D) Males PC3 vs PC4 and principal component analysis for entire dataset (E) PC1 vs PC2 and (F) PC3 vs PC4. AFR, African; AMR, American Admixed; EAS, East Asian; EUR, European; PC, Principal component; PCA, principal component analysis; SAPSC, South African Parkinson's Disease Study Collection; SAS, South Asian 4

Supplementary tables 5

Supplementary Table 1: Overview of the demographics of the study participants 5

Supplementary Table 2: List of the principal components used in each analysis regression 5

Supplementary Table 3: Inferred ancestry proportions for females versus males 5

Supplementary Table 4: Variants at chromosome-wide significance for the X chromosome wide association analyses 5

Banner Authors: Global Parkinson's Genetics Program 8

Supplementary methods

P-value threshold calculations

Number of independent tests= $\frac{number\ of\ SNPs^2}{sum}$

P-value threshold= $\frac{0.05}{number\ of\ independent\ tests}$

P-value threshold calculations for males

Number of independent tests = $\frac{1881820^2}{4277574334.2194767}$ = 827.8632318

P-value threshold = $\frac{0.05}{839.99897}$ = 6.039644966e-05

P-value threshold calculations for females

Number of independent tests = $\frac{1881820^2}{6816770379.713214}$ = 519. 490362

P-value threshold = $\frac{0.05}{519.490362}$ = 9.62481764e-05

Supplementary figures

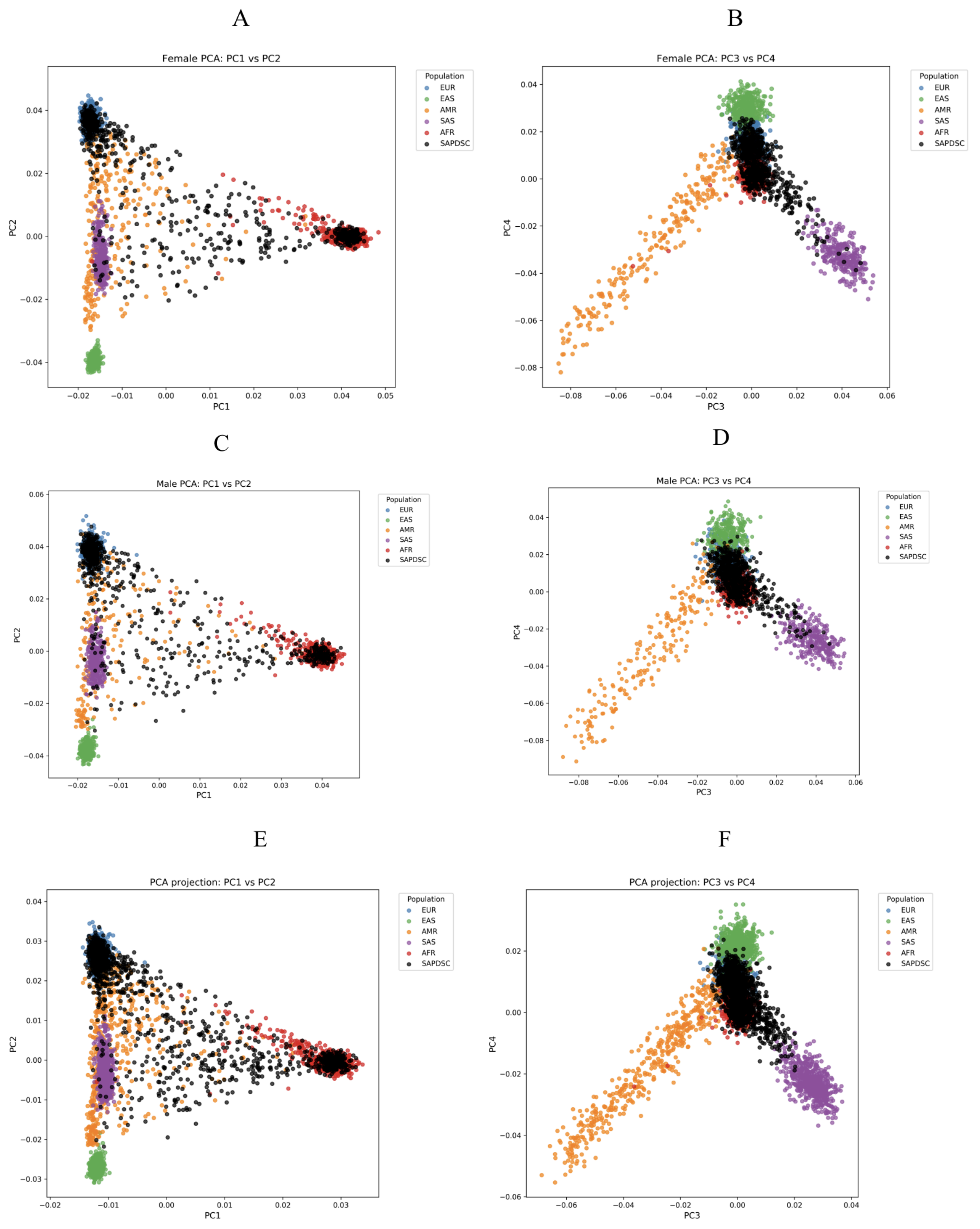

Supplementary Figure 1: Principal component analysis for (A) Females PC1 vs PC2, (B) Females PC3 vs PC4, (C) Males PC1 vs PC2, and (D) Males PC3 vs PC4 and principal component analysis for entire dataset (E) PC1 vs PC2 and (F) PC3 vs PC4. AFR, African; AMR, American Admixed; EAS, East Asian; EUR, European; PC, Principal component; PCA, principal component analysis; SAPDSC, South African Parkinson's Disease Study Collection; SAS, South Asian.

Supplementary tables

Supplementary Table 1: Overview of the demographics of the study participants

	Total participants	Mean (SD) age of recruitment	Number of males	Number of females
PD cases	658	66 (12)	380	278
Controls	737	45 (22)	346	391
Total	1395	55 (20)	726	669
Legend: SD, standard deviation				

Supplementary Table 2: List of the principal components used in each analysis regression

XWAS run	PCs included	Total PCs included	Other covariates included
Female	PC1, PC2, PC8, PC11, PC12, PC16, PC21, PC25, PC27, PC35, PC38, PC44, PC49	13	Age at recruitment
Male	PC1, PC2, PC3, PC9, PC11, PC19, PC22, PC23, PC27, PC33, PD45, PC47	12	Age at recruitment
Legend: Principal components for inclusion were determined using a stepwise regression. The XWAS analysis included the revelevant PCs and age as covariates. PCs, Principal components; XWAS, X chromosome wide association study			

Supplementary Table 3: Inferred ancestry proportions for females versus males

Sex	AFR	EAS	EUR	MALAY	NAMA	SAS
Female	0.1752	0.0001	0.5900	0.0468	0.1158	0.0713
Male	0.2020	0.0007	0.5291	0.0572	0.1441	0.0667
Legend: AFR, African; EUR, European; MALAY, Malay; Nama, Nama; SAS, South Asian						

Supplementary Table 4: Variants at chromosome-wide significance for the X chromosome wide association analyses

Variant ID	P-value	Nearest gene/ region	Consequence
**Sex-stratified XWAS: Males**			
rs1152049	3.27E-05	-	Intergenic variant
rs1775022	3.27E-05	-	Intergenic variant
rs1152054	3.27E-05	-	Intergenic variant

rs34517446	3.27E-05	-	Intergenic variant
rs200539602*	2.78E-05	*AL391666.1*	Intergenic variant
**Sex-stratified XWAS: Females**			
rs5914860	9.25E-05	*SPIN3*	Intron variant, NMD transcript variant
rs575574	9.19E-05	*FAAH2*	Intergenic variant
rs7892128	9.19E-05	*FAAH2*	Intergenic variant
rs543604	9.15E-05	*FAAH2*	Intergenic variant
rs473869	9.15E-05	*FAAH2*	Intergenic variant
rs4323611	8.70E-05	*FAAH2*	Intron variant
rs17252537	8.68E-05	*FAAH2*	Intergenic variant
rs482001	7.53E-05	*FAAH2*	Upstream gene variant, intron variant, non-coding transcript variant, downstream gene variant
rs555580	7.48E-05	*SPIN2A*	Intron variant, non-coding transcript variant
rs522751	7.48E-05	*SPIN2A*	Intron variant, non-coding transcript variant
rs56968572	7.17E-05	*FAAH2*	Upstream gene variant, intron variant, non-coding transcript variant
rs1591132	6.80E-05	*SPIN2A*	Intron variant, non-coding transcript variant, regulatory region variant
rs2516026	6.65E-05	*SPIN2A*	Intron variant, non-coding transcript variant
rs6521591	6.36E-05	*SPIN2A*	Intron variant, non-coding transcript variant
rs55905667	6.24E-05	*FAAH2*	Intergenic variant
rs5960899	5.88E-05	*SPIN2B*	Intergenic variant
rs2883203	5.88E-05	*SPIN2A*	Intronic variant, non-coding transcript variant
rs2499548	5.53E-05	*FAAH2*	Intergenic variant
rs2213509	5.49E-05	*FAAH2*	Intergenic variant
rs1100706	5.38E-05	*SPIN2A*	Intron variant, non-coding transcript variant
rs512119	5.38E-05	*SPIN2A*	Intron variant, non-coding transcript variant

rs556348	5.38E-05	*SPIN2A*	Intron variant, non-coding transcript variant
rs548158	5.38E-05	*SPIN2A*	Intron variant, non-coding transcript variant
rs6611612	3.88E-05	*FAAH2*	Intron variant
rs34207740	3.82E-05	*FAAH2*	Intron variant
rs2499550*	3.28E-05	*FAAH2*	Upstream gene variant
rs2516019	3.28E-05	*FAAH2*	Upstream gene variant
rs2499553	3.28E-05	*FAAH2*	Upstream gene variant
rs58045540*	2.42E-05	*AL844175.1*	Intergenic variant
**Meta-analysis**			
rs141026964*	5.73E-05	*RP11-492O8.1*	Intron variant, non-coding transcript variant
rs139302073	5.73E-05	*AL031073.2*	Intron variant, non-coding transcript variant
rs144268501	5.73E-05	-	Intron variant, non-coding transcript variant
rs115581157	3.13E-05	-	Intron variant, non-coding transcript variant, downstream variant
rs140715059*	1.68E-05	*RNA5SP516*	Intron variant, non-coding transcript variant, downstream variant
Legend: *, indicates the lead SNP/SNPs; CI, Confidence interval; N/A, Not applicable; NMD, nonsense-mediated mRNA decay pathway; XWAS, X chromosome wide association study			

Banner Authors: Global Parkinson's Genetics Program

Country	Name	Email address	Institution	City	State/Province (USA, Canada, Australia only)	Funders and Disclosures
**Algeria**	Yasser Mecheri	mecheriyasser@outlook.com	Centre Hospitalo-Universitaire Dr Benbadis Constantine	Constantine		Nothing to declare
**Algeria**	Bouchetara Mohamed Sofiane	neurobouchetara@yahoo.fr	Hospital university of Oran-Algeria	Oran		Nothing to declare
**Algeria**	Benhassine Traki	trakibenhassine@hotmail.com	Faculty of Biological Sciences, USTHB Bab Ezzouar, Algiers	Algiers		Nothing to declare
**Argentina**	Emilia M Gatto	egatto@ineba.net	Sanatorio de la Trinidad Mitre- INEBA	Buenos Aires		Nothing to declare
**Argentina**	Marcelo Kauffman	mkauffman@austral.edu.ar	Hospital JM Ramos Mejia	Buenos Aires		Nothing to declare
**Argentina**	Federico Capparelli	fcapparelli@fleni.org.ar	Centro de Educación Médica e Investigaciones Clínicas Norberto Quirno	Buenos Aires		Nothing to declare
**Argentina**	Maria Valentina Muller	mariavalentinamuller@gmail.com	Hospital General San Martin	La Plata		Nothing to declare
**Argentina**	Marcela Susana Tela	marcetela@gmail.com	Hospital Fernandez	Buenos Aires		Nothing to declare
**Argentina**	Adamec, Dario Sergio	darioadamec@yahoo.com.ar	HOSPITAL NACIONAL PROFESOR ALEJANDRO POSADAS	Buenos Aires		Nothing to declare
**Argentina**	Cesar Luis Avila	cesar.avila@fbqf.unt.edu.ar	CONICET-UNT	San Miguel de Tucumán		Nothing to declare
**Armenia**	Samson Khachatryan	drsamkhach@gmail.com	Somnus Neurology Clinic	Yerevan		Nothing to declare
**Armenia**	Zaruhi Tavadyan	zdtavadyan@gmail.com	Somnus Neurology Clinic	Yerevan		Nothing to declare

**Armenia**	Mariam Isayan	drmariamisayan@gmail.com	Somnus Neurology Clinic	Yerevan		Nothing to declare
**Australia**	Claire E Shepherd	c.shepherd@neura.edu.au	Neuroscience Research Australia	Sydney	New South Wales	The Sydney Brain Bank is located at and supported by Neuroscience Research Australia
**Australia**	Kishore Kumar	kkum4618@uni.sydney.edu.au	Garvan Institute of Medical Research and Concord Repatriation General Hospital	Darlinghurst	New South Wales	Paul Ainsworth Family Foundation
**Australia**	Melina Ellis	melina.ellis@health.nsw.gov.au	Concord Hospital	Concord	New South Wales	Nothing to declare
**Australia**	Miguel E. Rentería	miguel.renteria@qimrberghofer.edu.au	QIMR Berghofer Medical Research Institute	Herston	Queensland	The Australian Parkinson's Genetics Study is supported by the Shake It Up Australia Foundation and The Michael J. Fox Foundation for Parkinson's Research
**Australia**	Sulev Koks	sulev.koks@murdoch.edu.au	Murdoch University	Perth	Western Australia	Nothing to declare
**Australia**	Simon Rowe	s.rowe@neura.edu.au	Neuroscience Research Australia	Sydney	New South Wales	Nothing to declare
**Australia**	Dennis Yeow	d.yeow@neura.edu.au	Neuroscience Research Australia	Sydney	New South Wales	Nothing to declare
**Australia**	Carolyn Sue	c.sue@neura.edu.au	Neuroscience Research Australia	Sydney	New South Wales	Nothing to declare
**Australia**	Victor Flores Ocampo	victorfloresocampo@yahoo.com.mx	QIMR Berghofer Medical Research Institute	Brisbane	Queensland	Nothing to declare
**Australia**	Christine Wools	christine.wools@mh.org.au	Epworth hospital	Melbourne	Victoria	Nothing to declare
**Australia**	Keren Aliza	k.weiss@garvan.or	Garvan Institute of Medical Research	Sydney	New	Nothing to declare

	Weiss	g.au			South Wales	
**Australia**	Sue-Faye Siow	suefaye.siow@health.nsw.gov.au	Royal North Shore Hospital	Sydney	New South Wales	Nothing to declare
**Australia**	Ryan L Davis	ryan.davis@sydney.edu.au	University of Sydney	Sydney	New South Wales	Nothing to declare
**Australia**	Amanda Willis	a.willis@garvan.org.au	Garvan Institute of Medical Research	Sydney	New South Wales	Nothing to declare
**Australia**	Steven He	s.he@garvan.org.au	Garvan Institute of Medical Research	Sydney	New South Wales	Nothing to declare
**Australia**	Robert Arthur Wilcox	Robert.Wilcox@sa.gov.au	Flinders Medical Centre	Bedford Park	South Australia	Nothing to declare
**Australia**	Denise Howting	denise.howting@perron.uwa.edu.au	Perron Institute for Neurological and Translational Science	Nedlands	Western Australia	Nothing to declare
**Australia**	Jack David Price	jack.price@perron.uwa.edu.au	Perron Institute	Perth	Western Australia	Nothing to declare
**Australia**	Pak Leng Cheong	pak.cheong@health.nsw.gov.au	Sydney Local Health District	Sydney	New South Wales	Nothing to declare
**Australia**	Michel Tchan	michel.tchan@health.nsw.gov.au	Westmead Hospital	Westmead	New South Wales	Nothing to declare
**Australia**	Mary-Anne Young	m.young@garvan.org.au	MonoPD	Sydney	New South Wales	Nothing to declare
**Australia**	Catriona Mclean	c.mclean@alfred.org.au	Florey neuroscience	Melbourne	Victoria	Nothing to declare

**Australia**	Nicholas G. Martin	nick.martin@qimrb.edu.au	QIMR Berghofer Medical Research Institute	Brisbane	Queensland	Nothing to declare
**Australia**	Hugo Morales Briceño	hugo.moralesbriceno@health.nsw.gov.au	Westmead Hospital	Sydney	New South Wales	Nothing to declare
**Australia**	Thomas Kimber	thomas.kimber@sa.gov.au	Central Adelaide Local Health Network	Adelaide	South Australia	Nothing to declare
**Australia**	Kathy H. C. Wu	kathy.wu@svha.org.au	St Vincent's Hospital Sydney	Darlinghurst		Nothing to declare
**Australia**	John O'Sullivan	john.osullivan@uq.edu.au	University of Queensland	Brisbane		Nothing to declare
**Australia**	Lewis M Singleton	lewis.singleton@perron.uwa.edu.au	Perron Institute of Neurological and Translational Science	Perth		Nothing to declare
**Australia**	Laura Ivete Rudaks	laura.rudaks@gmail.com	Concord Repatriation General Hospital	Sydney		Nothing to declare
**Australia**	Luis M. García-Marín	garciamarinlm@gmail.com	QIMR Berghofer	Brisbane		Nothing to declare
**Austria**	Alexander Zimprich	alexander.zimprich@meduniwien.ac.at	Medical University Vienna Austria	Vienna		Nothing to declare
**Azerbaijan**	Kanan Jafarov	kcaferov1975@gmail.com	Istanbul Klinik	Baku		Nothing to declare
**Azerbaijan**	Kenan Ceferov	kcaferov1975@gmail.com	Istanbul clinic Movement disorders center	Baku		Nothing to declare
**Bangladesh**	Imran Sarker	imran37mmc@gmail.com	National Institute of Neurosciences and Hospital	Dhaka		Nothing to declare
**Belgium**	David Crosiers	david.crosiers@uantwerpen.be	University of Antwerp	Antwerp		Nothing to declare
**Brazil**	Artur F. Schumacher-Schuh	aschuh@hcpa.edu.br	Universidade Federal do Rio Grande do Sul / Hospital de Clínicas de Porto Alegre	Porto Alegre		Nothing to declare
**Brazil**	Carlos Rieder	carlosrieder@gmail.com	Federal University of Health Sciences of Porto Alegre	Porto Alegre		Nothing to declare

**Brazil**	Paula Saffie Awad	paula.saffie@clinicasantamaria.cl	Universidade Federal do Rio Grande do Sul	Porto Alegre		Global Parkinson's Genetics Program
**Brazil**	Vitor Tumas	tumasv@fmrp.usp.br	University of São Paulo	São Paulo		Nothing to declare
**Brazil**	Sarah Camargos	sarahcamargos@ufmg.br	Universidade Federal de Minas Gerais	Belo Horizonte		Nothing to declare
**Brazil**	Lucas Faria Costa	farialucas13@gmail.com	Universidade Federal de Minas Gerais	Belo Horizonte		Nothing to declare
**Brazil**	Pedro Braga Neto	pbraganeto@ufc.br	Federal University of Ceará	Fortaleza		Nothing to declare
**Canada**	Oury Monchi	oury.monchi@umontreal.ca	Institut universitaire de gériatrie de Montréal	Montreal	Quebec	CIHR, Brain Canada, Parkinson Canada
**Canada**	Edward Fon	ted.fon@mcgill.ca	McGill University	Montreal	Quebec	Nothing to declare
**Canada**	Robert Thibault	rthibault@parkinsonsroadmap.org	Aligning Science Across Parkinson's	Vancouver	British Columbia	Nothing to declare
**Canada**	Ziv Gan-Or	ziv.gan-or@mcgill.ca	McGill University	Montreal	Quebec	Nothing to declare
**Canada**	Meron Teferra	meron.teferra@mcgill.ca	McGill University	Montreal	Quebec	Nothing to declare
**Canada**	Anthony Lang	Anthony.Lang@uhnresearch.ca	University of Toronto	Toronto	Ontario	Nothing to declare
**Canada**	Konstantin Senkevich	konstantin.senkevich@mcgill.ca	McGill University	Montreal	Quebec	Nothing to declare
**Chile**	Marcelo Miranda	Directiva@fundaciondiagnosis.cl	Fundación Diagnosis	Santiago		Nothing to declare
**Chile**	Maria Leonor Bustamante	mbustamante@uchile.cl	Faculty of Medicine Universidad de Chile	Santiago		Nothing to declare
**Chile**	Juan Cristobal Nuñez	jc.nunezf@gmail.com	Universidad de Chile - Clínica Alemana Santiago	Santiago		Nothing to declare
**Chile**	Boris Lucero	bluceroucm@gmail.com	Universidad Católica del Maule	Talca		Nothing to declare

**Chile**	Alicia Colombo	acolombo@hcuch.cl	University of Chile	Santiago		Nothing to declare
**Chile**	Maria Teresa Muñoz Personal	mtmunoz@uchile.cl	Universidad de Chile	Santiago		Internal Research Fund – Occupational pesticide exposure and Parkinson's disease risk in older Chilean adults from the LARGE-PD cohort, School of Public Health, Universidad de Chile.<br><br>FONDECYT Regular 1240899 – Environmental exposure to pesticides and neurobehavioral and neurophysiological effects in adults from rural communities in the Maule Region, Chile
**Chile**	Eduardo Pérez Palma	eperezpalma@gmail.com	Universidad del Desarrollo	Santiago		Nothing to declare
**Chile**	Pedro Chana-Cuevas	pchana@cetram.org	Universidad de Santiago de Chile	Santiago		Nothing to declare
**Chile**	Ana Belen Miranda Cortes	anamirandacortes@gmail.com	Fundación Diagnosis	Santiago		Nothing to declare
**Chile**	María Eugenia Contreras Pinto	mecontreraspinto@gmail.com	Hospital San Juan de Dios	La serena		Nothing to declare
**Chile**	Francisca Canals	mfcanals@gmail.com	Inmov	Santiago		Nothing to declare
**Chile**	Benjamín Pizarro-Galleguillos	benjaminpizarro@ug.uchile.cl	Universidad de Chile	Santiago		Nothing to declare
**Chile**	Patricio	patricioolguin@uch	Universidad de Chile, Facultad de Medicina	Santiago		Nothing to declare

	Alejandro Olguín Aguilera	ile.cl				
**Chile**	Elias Fernandez-Toledo	elias.mft@gmail.com	University of Concepción	Concepcion		Nothing to declare
**Chile**	Benjamin Pizarro Galleguillos	benjaminpizarro@ug.uchile.cl	Centro de Imagenología, Hospital Clínico Universidad de Chile	Santiago		Nothing to declare
**China**	Beisha Tang	bstang7398@csu.edu.cn	Central South University	Changsha		Nothing to declare
**China**	Huifang Shang	shanghuifang@scu.edu.cn	West China Hospital Sichuan University	Chengdu		Nothing to declare
**China**	Jifeng Guo	guojifeng2003@163.com	Xiangya Hospital	Changsha		Nothing to declare
**China**	Piu Chan	pbchan@hotmail.com	Capital Medical University	Beijing		Nothing to declare
**China**	Wei Luo	weiluo@zju.edu.cn	Zhejiang University	Hangzhou		Nothing to declare
**China**	Zhenhua Liu	liuzhenhua@csu.edu.cn	Xiangya Hospital, Central South University	Changsha		Nothing to declare
**China**	Germaine Hiu-Fai Chan	Chf862@ha.org.hk	Queen Elizabeth Hospital	Kowloon		Nothing to declare
**China**	Nancy Ip	boip@ust.hk	The Hong Kong University of Science and Technology	Kowloon		Nothing to declare
**China**	Nelson Yuk-Fai Cheung		Queen Elizabeth Hospital	Kowloon		Nothing to declare
**China**	Phillip Chan	philipch.chan@polyu.edu.hk	The Hong Kong University of Science and Technology	Kowloon		Nothing to declare
**China**	Xiaopu Zhou	xpzhou@ust.hk	The Hong Kong University of Science and Technology	Kowloon		Nothing to declare
**Colombia**	Gonzalo Arboleda	gharboledab@unal.edu.co	Universidad Nacional de Colombia	Bogotá		Nothing to declare
**Colombia**	Jorge Orozco	jorge.orozco@fvl.org.co	Fundación Valle del Lili	Santiago De Cali		Nothing to declare

**Colombia**	David Antonio Pineda-Salazar	dapinedasas@gmail.com	GRUPO DE NEUROCIENCIAS DE ANTIOQUIA (GNA)	Medellín		Nothing to declare
**Colombia**	Beatriz Munoz Ospina	beatriz.munoz@fvl.org.co	Fundacion Valle del Lili	Santiago De Cali		Nothing to declare
**Colombia**	Tatiana Lopez-Gonzalez	ntlopezg@gmail.com	Universidad Nacional de Colombia	Bogotá		Nothing to declare
**Colombia**	Carlos Velez-Pardo	calberto.velez@udea.edu.co	Universidad de Antioquia	Medellín		Nothing to declare
**Colombia**	Marlene Jimenez-Del-Rio	marlene.jimenez@udea.edu.co	Universidad de Antioquia	Medellín		Nothing to declare
**Colombia**	Sonia Moreno Masmela	sonia.moreno@gna.org.co	Universidad de Antioquia	Medellín		Nothing to declare
**Costa Rica**	Alvaro Hernandez	ahernandez@williamblair.com	University of Costa Rica	San Jose		Nothing to declare
**Denmark**	Per Borghammer	borghammer@clin.au.dk	Aarhus University	Aarhus		Nothing to declare
**Egypt**	Mohamed Salama	mohamed-salama@aucegypt.edu	The American University in Cairo	Cairo		The AUC/ ASRT/ DAAD
**Egypt**	Walaa A. Kamel	Walaaneuro@med.bsu.edu.eg	Beni-Suef University	Beni Suef		Nothing to declare
**El Salvador**	Tatiana Ascencio	tatianaascencio@gmail.com	Dr. Andres Bello university	San Salvador		Nothing to declare
**El Salvador**	Oscar Peña-Rodas	oscar.penarodas@gmail.com	Universidad Dr Andrés Bello	San Salvador		Nothing to declare
**El Salvador**	Susana Lissette Peña Martínez	dra.susanapneuro@gmail.com	UNAB	San Salvador		Nothing to declare
**Ethiopia**	Yared Z.	yared.zenebe@aau.	Addis Ababa University	Addis Ababa		Nothing to declare

	Zewde	edu.et				
**France**	Alexis Brice	alexis.brice@icm-institute.org	Paris Brain Institute	Paris		Nothing to declare
**France**	Jean-Christophe Corvol	jean-christophe.corvol@aphp.fr	Sorbonne Université	Paris		Nothing to declare
**France**	Mari Vidailhet	marie.vidailhet@aphp.fr	Salpêtriere Hospital (AP-HP), Sorbonne Université	Paris		Nothing to declare
**France**	Mathieu Anheim	mathieu.anheim@chru-strasbourg.fr	University Hospital of Strasbourg, Strasbourg, France	STRASBOURG		Nothing to declare
**France**	Yves Agid	yves.agid@icm-institute.org	Paris Brain Institute	Paris		Nothing to declare
**France**	Louise-Laure Mariani	louise-laure.mariani@icm-institute.org	Paris Brain Institute - Sorbonne University	Paris		Nothing to declare
**France**	Alexandra Durr	alexandra.durr@icm-institute.org	Paris Brain Institute	Paris		Nothing to declare
**France**	Rascol	olivier.rascol@univ-tlse3.fr	université Toulouse	toulouse		Nothing to declare
**France**	Ory Magne Fabienne	ory.f@chu-toulouse.fr	chu toulouse	toulouse		Nothing to declare
**France**	Suzanne Lesage	laly.lesage@gmail.com	Paris Brain Institute (ICM)	Paris		Nothing to declare
**France**	Defebvre Luc	luc.defebvre@chu-lille.fr	CHU Lille	Lille		Nothing to declare
**France**	Tesson Christelle	christelle.tesson@icm-institute.org	Institut du Cerveau-Paris Brain Institute-ICM	Paris		Nothing to declare
**France**	Philippe Damier	philippe.damier@univ-nantes.fr	Nantes Université	Nantes		Nothing to declare
**France**	François Tison	francois.tison@chu-bordeaux.fr	University of Bordeaux, France	Bordeaux		Nothing to declare
**France**	Stéphane Thobois	stephane.thobois@chu-lyon.fr	Hospices civils de Lyon, Hopital Neurologique Pierre Wertheimer	BRON		Nothing to declare
**France**	Jean-Luc Houeto	jean-luc.houeto@unilim.fr	Limoges University Hospital	Limoges		Nothing to declare
**France**	Brefel	christine.brefel-cou	CHU Toulouse	TOULOUSE		Nothing to declare

	Courbon Christine	rbon@univ-tlse3.fr				
**France**	Sara Sambin	sara.sambin@icm-institute.org	CIC Neurosciences ,Paris Brain institute	Paris		Nothing to declare
**France**	Aymeric Lanore	lanore.aymeric@gmail.com	Paris Brain Institute	Paris		Nothing to declare
**France**	NS-PARK Consortium	/	/	/		/
**Georgia**	Mariam Kekenadze	mariam.kekenadze@gmail.com	Tbilisi State Medical University	Tbilisi		Nothing to declare
**Georgia**	Irine Khatiashvili	irenekhat@yahoo.com	S. Khechinashvili University Hospital	Tbilisi		Nothing to declare
**Georgia**	Maia Beridze	postdoc2002@gmail.com	Tbilisi State Medical University	Tbilisi		Nothing to declare
**Georgia**	Sophia Sopromadze	ssopromadze@yahoo.com	Ivane Javakhishvili Tbilisi State University	Tbilisi		Nothing to declare
**Georgia**	Irine Khatiashvilli	irenekhat@yahoo.com	Ivane Javakhishvili Tbilisi State University	Tbilisi		Nothing to declare
**Georgia**	Mariam Mshvenieradze	mariammshvenieridze5@gmail.com	Ivane Javakhishvili Tbilisi State University	Tbilisi		Nothing to declare
**Georgia**	Marika Megrelishvili	marikamegre@gmail.com	Ilia State University	Tbilisi		Nothing to declare
**Georgia**	Alexander Tsiskaridze	alexander.tsiskaridze@tsu.ge	Ivane Javakhishvili Tbilisi State University	Tbilisi		Nothing to declare
**Germany**	Ana Westenberger	ana.westenberger@uni-luebeck.de	University of Lübeck	Lübeck		Nothing to declare
**Germany**	Anastasia Illarionova	anastasia.illarionova@dzne.de	Deutsches Zentrum für Neurodegenerative Erkrankungen	Göttingen		Nothing to declare
**Germany**	Brit Mollenhauer	brit.mollenhauer@med.uni-goettingen.de	University Medical Center Göttingen	Göttingen		Nothing to declare

**Germany**	Christine Klein	christine.klein@neuro.uni-luebeck.de	University of Lübeck	Lübeck		CK serves as a medical Advisor to Centogene on genetic testing reports in the field of movement disorders, except Parkinson's disease, and is a member of the Scientific Advisory Board of Retromer Therapeutics
**Germany**	Eva-Juliane Vollstedt	jule.vollstedt@neuro.uni-luebeck.de	University of Lübeck	Lübeck		Nothing to declare
**Germany**	Franziska Hopfner	franziska.hopfner@med.uni-muenchen.de	Department of Neurology, University Hospital, LMU Munich	Munich		Nothing to declare
**Germany**	Günter Höglinger	Guenter.Hoeglinger@med.uni-muenchen.de	Department of Neurology, University Hospital, LMU Munich	Munich		Nothing to declare
**Germany**	Harutyun Madoev	harutyun.madoev@neuro.uni-luebeck.de	University of Lübeck	Lübeck		Nothing to declare
**Germany**	Joanne Trinh	joanne.trinh@neuro.uni-luebeck.de	University of Lübeck	Lübeck		Nothing to declare
**Germany**	Katja Lohmann	katja.lohmann@neuro.uni-luebeck.de	University of Lübeck	Lübeck		Nothing to declare
**Germany**	Manu Sharma	manu.sharma@uni-tuebingen.de	University of Tubingen	Tübingen		Dr. Sharma is further funded by the Michael J Fox Foundation, USA Genetic Diversity in PD Program: GAP-India Grant ID: 009411.
**Germany**	Sergiu Groppa	segroppa@uni-mainz.de	University of Mainz	Mainz		Nothing to declare
**Germany**	Thomas Gasser	thomas.gasser@uni-tuebingen.de	University of Tübingen	Tübingen		Nothing to declare
**Germany**	Zih-Hua Fang	Zih-Hua.Fang@dzne.de	The German Center for Neurodegenerative Diseases	Göttingen		Nothing to declare

**Germany**	Karl Heilbron	kheilbro@broadinstitute.org	Charité - Universitätsmedizin Berlin	Berlin		Former employee of 23andMe, Inc. Owns stock and/or stock options in 23andMe, Inc.
**Germany**	Wenhua Sun	wenhua.sun@uni-tuebingen.de	University of Teubingen	Tübingen		Nothing to declare
**Germany**	Inke König	inke.koenig@uni-luebeck.de	University of Lübeck	Lübeck		Nothing to declare
**Germany**	Daniela Berg	daniela.berg@uksh.de	University Medical Center Schleswig-Holstein	Lübeck		Nothing to declare
**Germany**	Bernhard Haslinger	bernhard.haslinger@tum.de	Technical University of Munich	Munich		Nothing to declare
**Germany**	Teresa Kleinz	teresa.kleinz@uni-luebeck.de	University of Lübeck	Lübeck		Nothing to declare
**Germany**	Norbert Brüggemann	norbert.brueggemann@uni-luebeck.de	University of Lübeck	Lübeck		Dr. Brüggemann received honaria from Abbvie, Esteve, Ipsen, Merz, Teva and Zambon
**Germany**	Konstantin Kufer	Konstantin.Kufer@dzne.de	German Centre for Neurodegenerative Diseases (DZNE) / University Hospital Bonn	Bonn		Nothing to declare
**Germany**	Antonia Maria Buchal	Antonia.Buchal@ukbonn.de	University Hospital Bonn	Bonn		Nothing to declare
**Germany**	Matthias Höllerhage	hoellerhage.matthias@mh-hannover.de	Hannover Medical School	Hannover		Nothing to declare
**Germany**	Florian Wegner	wegner.florian@mh-hannover.de	Hannover Medical School	Hannover		Nothing to declare
**Germany**	Nils Schroeter	Nils.Schroeter@uks.eu	UKS, University of Saarland	Homburg and Mainz		Nothing to declare
**Germany**	Kathrin Brockmann	kathrin.brockmann@uni-tuebingen.de	University of Tübingen	Tübingen		KB has received research funding from the Michael J. Fox Foundation for Parkinson's Research, the German Society for Parkinson DPG, the Health Forum Baden Wuerttemberg, the Else Kröner Fresenius Stiftung,

						the University of Tuebingen, and from the German Research Foundation DFG. KB is a consultant for F. Hoffmann-La Roche Ltd., Vanqua Bio, and the Michael J. Fox Foundation for Parkinson's Research and has received speaker honoraria from Abbvie, Lundbeck, UCB and Zambon.
**Germany**	Isabel Wurster	isabel.wurster@med.uni-tuebingen.de	University of Tübingen	Tübingen		Nothing to declare
**Germany**	Theresa Lüth	t.lueth@uni-luebeck.de	University of Lübeck	Lübeck		Nothing to declare
**Germany**	Christian Beetz	christian.beetz@centogene.com	CENTOGENE	Rostock		Nothing to declare
**Germany**	Krishnakumar Kandaswamy	krishna.kandaswamy@centogene.com	Centogene GmBH	Berlin		Nothing to declare
**Germany**	Eva Schäffer	eva.schaeffer@uksh.de	Kiel University	Kiel		Nothing to declare
**Germany**	Kirsten Zeuner	kirsten.zeuner@uksh.de	Kiel University	Kiel		Nothing to declare
**Germany**	Gregor Kuhlenbäumer	gregor.kuhlenbaeumer@uksh.de	Kiel University	Kiel		Nothing to declare
**Germany**	Peter Bauer	peter.bauer@centogene.com	Centogene GmbH	Rostock		Nothing to declare
**Germany**	Martin Klietz	klietz.martin@mh-hannover.de	Hannover Medical School	Hannover		Nothing to declare
**Germany**	Carolin Gabbert	c.gabbert@uni-luebeck.de	University of Lübeck	Lübeck		Nothing to declare
**Germany**	Alexander	alexander.balck@u	University of Lübeck	Lübeck		Nothing to declare

	Balck	ni-luebeck.de				
**Ghana**	Albert Akpalu	alakpalu@ug.edu.gh	University of Ghana Medical School	Accra		Nothing to declare
**Ghana**	Momodou Cham	modoufa@gmail.com	Richard Novati Catholic Hospital, Catholic Health Service Trust	Accra		Nothing to declare
**Ghana**	Vida Obese	nadwos@hotmail.com	Kwame Nkrumah University of Science and Technology	Kumasi		Nothing to declare
**Greece**	Georgia Xiromerisiou	geoksirom@med.uth.gr	University of Thessaly	Volos		Nothing to declare
**Greece**	Georgios Hadjigorgiou	G.Hadjigeorgiou@euc.ac.cy	University of Thessaly	Volos		Nothing to declare
**Greece**	Ioannis Dagklis	iadaglis@phys.uoa.gr	Aristotle University of Thessaloniki	Thessaloniki		Nothing to declare
**Greece**	Ioannis Tarnanas	ioannis.tarnanas@gbhi.org	Ionian University	Corfu		Nothing to declare
**Greece**	Leonidas Stefanis	lstefanis@bioacademy.gr	Biomedical research Foundation of the Academy of Athens	Athens		PPMI2 (funded by MJFF), ALAMEDA (H2020 grant), funding by HFRI
**Greece**	Maria Stamelou	mstamelou@hygeia.gr	Diagnostic and Therapeutic Centre HYGEIA Hospital	Marousi		The non-profit organisation for scientific research in Parkinson's disease and related disorders
**Greece**	Efthymios Dadiotis	edar@med.uth.gr	University of Thessaly	Volos		Nothing to declare
**Greece**	Tsamis Konstantinos	ktsamis@uoi.gr	University of Ioannina	Ioannina		Honoraria and research funding: Sanofi, Genesis, Abbvie, Lavipharm, Medochemie, IPSEN, Bennett, PD Neurotechnology
**Greece**	Konitsiotis Spyridon	skonitso@uoi.gr	University of Ioannina	Ioannina		Honoraria and research funding: Novartis, Sanofi, BMS, TEVA, Merck, Genesis, Innovis, Viatris, Abbvie, Lundbeck, Angelini, UCB, Lavipharm, Φαρμασερβ, Elpen, Roche, Sandoz,

						Medtronic, Medison, AstraZeneca, ITF, IPSEN, PD Neurotechnology
**Greece**	Iro Boura	boura.iro@gmail.com	University of Crete	Heraklion		University of Crete, Changeing-Excellence Hub, AbbVie
**Greece**	Maria	marizmak711@gmail.com	HYGEIA Hospital	Athens		Nothing to declare
**Greece**	Lina Florentin	lflorentin@alab.gr	HYGEIA Hospital	Athens		Nothing to declare
**Greece**	Maria Makrygianni	marizmak711@gmail.com	HYGEIA Hospital	Athens		Nothing to declare
**Greece**	Foivos S. Kanellos	f.kanellos@uoi.gr	University of Ioannina	Ioannina		Nothing to declare
**Greece**	Cleanthe Spanaki	kliospanaki@gmail.com	University of Crete	Heraklion		C.S. has received honoraria for lecturing, advisory fees, educational grants, and travel grants from ITF Hellas, Innovis, Merck Serono, AbbVie, CSL Behring, Roche and Teva. She received research funding from the Greek National Precision Medicine Network, the European Union's Horizon 2020 research innovation program under the Marie Sklodowska-Curie grant agreement No 101007926 and from the European Union's program Excellence Hubs - HORIZON-WIDERA-2022-ACCESS-04-01 under grant agreement No. 101087071.
**Honduras**	Alex Medina	alexmedina84@hotmail.com	Hospital San Felipe	Tegucigalpa		Nothing to declare

**Honduras**	Evelin Álvarez Herrera	evelynah.ea@gmail.com	Universidad Tecnológica Centroamericana (UNITEC)	Tegucigalpa		Nothing to declare
**Honduras**	Heike Hesse Joya	hhesse.neurologia@gmail.com	Universidad Tecnológica Centroamericana UNITEC	Tegucigalpa		Nothing to declare
**Honduras**	Reyna M. Durón	reynamduron@gmail.com	Universidad Tecnológica Centroamericana	Tegucigalpa		Nothing to declare
**Honduras**	Glenda Oliva Fuentes	glenda.oliva13@gmail.com	Fundación Lucas para la Salud	Tegucigalpa		Nothing to declare
**Honduras**	Eduardo Jose Ponce Murillo	eduardomurillo1795@gmail.com	Universidad Tecnológica Centroamericana	Tegucigalpa		Nothing to declare
**Iceland**	Kari Stefansson	kstefans@decode.is	deCODE genetics/Amgen Inc., Reykjavik, Iceland Faculty of Medicine, University of Iceland, Reykjavik, Iceland	Reykjavik		employee of deCODE genetics/Amgen inc
**Iceland**	Hreinn Stefansson	hreinn@decode.is	deCODE genetics/Amgen Inc., Reykjavik, Iceland Faculty of Medicine, University of Iceland, Reykjavik, Iceland	Reykjavik		employee of deCODE genetics/Amgen inc
**Iceland**	Vala Palmadottir	valakp@landspitali.is	deCODE genetics/Amgen Inc., Reykjavik, Iceland Faculty of Medicine, University of Iceland, Reykjavik, Iceland	Reykjavik		employee of deCODE genetics/Amgen inc
**Iceland**	Astros Th. Skuladottir	astross@decode.is	deCODE genetics/Amgen Inc., Reykjavik, Iceland Faculty of Medicine, University of Iceland, Reykjavik, Iceland	Reykjavik		employee of deCODE genetics/Amgen inc
**India**	Asha Kishore	Asha.kishore@asterdmhealthcare.com	Aster Medcity	Kochi		Michael J. Fox Foundation
**India**	Divya Kp	drdivyakp@sctimst.ac.in	Sree Chitra Tirunal Institute for Medical Sciences and Technology	Thiruvananthapuram		Nothing to declare
**India**	Pramod Pal	palpramod@nimhans.ac.in	National Institute of Mental Health & Neurosciences	Bengaluru		Nothing to declare
**India**	Prashanth Lingappa Kukkle	drprashanth.lk@movementdisordersclinic.com	Manipal Hospital	Delhi		Nothing to declare
**India**	Roopa Rajan	rooparajan@aiims.edu	All India Institute of Medical Sciences	Delhi		Nothing to declare
**India**	Rupam Borgohain	b_rupam@hotmail.com	Nizam's Institute Of Medical Sciences	Hyderabad		Nothing to declare
**Iran**	Mehri Salari	mehri.salari@gmail	Shahid Beheshti University of Medical Science	Tehran		Nothing to declare

		.com				
**Israel**	Tamara Shiner	tamarsh@tlvmc.gov.il	Tel Aviv Sourasky Medical Center	Tel Aviv-Yafo		Nothing to declare
**Israel**	Avner Thaler	avnert@tlvmc.gov.il	Tel Aviv Sourasky Medical Center	Tel Aviv-Yafo		Nothing to declare
**Israel**	Noa Bregman	noabr@tlvmc.gov.il	Tel Aviv Medical Center	Tel Aviv-Yafo		Nothing to declare
**Italy**	Andrea Quattrone	an.quattrone@icloud.com	Magna Græcia University of Catanzaro	Catanzaro		Nothing to declare
**Italy**	Enza Maria Valente	enzamaria.valente@unipv.it	University of Pavia	Pavia		Nothing to declare
**Italy**	Grazia Annesi	grazia.annesi@cnr.it	National Research Council	Cosenza		Nothing to declare
**Italy**	Lucilla Parnetti	lucilla.parnetti@unipg.it	University of Perugia	Perugia		Nothing to declare
**Italy**	Micol Avenali	micol.avenali@mondino.it	University of Pavia	Pavia		Nothing to declare
**Italy**	Monica Gagliardi	monica.gagliardi@unicz.it	Magna Graecia University	Catanzaro		Nothing to declare
**Italy**	Tommaso Schirinzi	tommaso.schirinzi@opbg.net	University of Rome Tor Vergata	Rome		Nothing to declare
**Italy**	Caterina Galandra	caterina.galandra@mondino.it	IRCCS Mondino Foundation	Pavia		Nothing to declare
**Italy**	Anna De Rosa	anna.derosa1@unina.it	University of Naples Federico II	Naples		Nothing to declare
**Italy**	Rosangela Ferese	ferese.rosangela@gmail.com	IRCCS Neuromed	Pozzilli		Nothing to declare
**Italy**	Jolanda Buonocore	jolandabuonocore@gmail.com	Magna Graecia University	Catanzaro		Nothing to declare
**Italy**	Radha Procopio	radha.procopio@unicz.it	Magna Graecia University	Catanzaro		Nothing to declare
**Italy**	Ilaria Palmieri	ilaria.palmieri01@universitadipavia.it	IRCCS Mondino Foundation	Pavia		Nothing to declare
**Italy**	Michele	michele.terzaghi@	University of Pavia	Pavia		Nothing to declare

	Terzaghi	mondino.it				
**Japan**	Manabu Funayama	funayama@juntendo.ac.jp	Juntendo University	Tokyo		Nothing to declare
**Japan**	Nobutaka Hattori	nhattori@juntendo.ac.jp	Juntendo University faculty of medicine	Tokyo		Nothing to declare
**Japan**	Tomotaka Shiraishi	shiraishito@jikei.ac.jp	Jikei University School of Medicine	Tokyo		Nothing to declare
**Japan**	Kensuke Daida	k-daida@juntendo.ac.jp	Juntendo University	Bunkyō		Nothing to declare
**Kazakhstan**	Altynay Karimova	neurokz@mail.ru	Institute of Neurology and Neurorehabilitation	Almaty		Nothing to declare
**Kazakhstan**	Gulnaz Kaishibayeva	Neurology_institute@mail.ru	Institute of Neurology and Neurorehabilitation	Almaty		Nothing to declare
**Kazakhstan**	Aigerim Utegenova	87012226598@mail.ru	West Kazakhstan Marat Ospanov State Medical University	Aktobe		Nothing to declare
**Kazakhstan**	Vadim Akhmetzhanov	vadiman49@gmail.com	Medline medical center	Astana		Nothing to declare
**Kazakhstan**	Seitzhan Aidarov	seitzhanaidarov@gmail.com	National Center for Neurosurgery	Astana		Nothing to declare
**Kazakhstan**	Tautanova Raushan	raushanat@gmail.com	Astana Medical University	Astana		Nothing to declare
**Kazakhstan**	Dinara Alzhanova	dinara.alzhanowa@yandex.ru	Astana Medical University	Astana		Nothing to declare
**Kazakhstan**	Zhanybek Myrzayev	myrzaevjanik@gmail.com	International University of Postgraduate Education	Almaty		Nothing to declare
**Kazakhstan**	Saltanat Abdraimova	abdraimovasaltanat06@gmail.com	South Kazakhstan Medical Academy	Shymkent		Nothing to declare
**Kazakhstan**	Nazira Zharkinbekova	nazirazhar@mail.ru	South Kazakhstan Medical Academy	Shymkent		South Kazakhstan Medical academy
**Kazakhstan**	Chingiz Shashkin	chingizshashkin@gmail.com	International Research Institute of Postgraduate Education	Almaty		Nothing to declare

**Kazakhstan**	Guzel Shiderova	guzelshiderova@gmail.com	Institute of Neurology and Neurorehabilitation	Almaty		Nothing to declare
**Kazakhstan**	Bagzhan Syzdykova	syzdykova-75@mail.ru	Astana Medical University	Astana		Nothing to declare
**Kazakhstan**	Aigul. P. Yermagambetova	aiguler72@mail.ru	West Kazakhstan Marat Ospanov Medical University	Aktobe		Nothing to declare
**Kazakhstan**	Alima A. Khamidulla	alima.khamidulla@gmail.com	West Kazakhstan Marat Ospanov Medical University	Aktobe		Nothing to declare
**Kazakhstan**	Zhanylsyn U.Urasheva	zhanylsyn.urasheva90@gmail.com	West Kazakhstan Marat Ospanov Medical University	Aktobe		Nothing to declare
**Kazakhstan**	Gulnar B. Kabdrakhmanova	nevrolog.ufa@gmail.com	West Kazakhstan Marat Ospanov Medical University	Aktobe		Nothing to declare
**Kazakhstan**	Talgat Khaibullin	htn2006@mail.ru	Semey Medical University	Semey		Nothing to declare
**Kazakhstan**	Altynay Talgatkyzy	neurokz@mail.ru	Semey Medical University	Semey		Nothing to declare
**Kyrgyzstan**	Cholpon Shambetova	cholpon.shambetova@gmail.com	Kyrgyz State Medical Academy	Bishkek		Nothing to declare
**Luxembourg**	Rejko Krüger	rejko.krueger@uni.lu	University of Luxembourg	Esch-sur-Alzette		Nothing to declare
**Luxembourg**	Patrick May	patrick.may@uni.lu	University of Luxembourg	Esch-sur-Alzette		Nothing to declare
**Malaysia**	Ai Huey Tan	tan.aihuey@ummc.edu.my	University of Malaya	Kuala Lumpur		Nothing to declare
**Malaysia**	Azlina Ahmad-Annuar	azlina_aa@um.edu.my	University of Malaya	Kuala Lumpur		Nothing to declare
**Malaysia**	Mohamed Ibrahim Norlinah	norlinah@ppukm.ukm.edu.my	Universiti Kebangsaan Malaysia	Selangor		Nothing to declare
**Malaysia**	Nor Azian Abdul	nor_azian@ppukm.ukm.edu.my	UKM Medical Molecular Biology Institute	Kuala Lumpur		Nothing to declare

	Murad					
**Malaysia**	Shahrul Azmin	shahrulazmin@hotmail.com	Universiti Kebangsaan Malaysia Medical Centre	Kuala Lumpur		Nothing to declare
**Malaysia**	Shen-Yang Lim	sylim@ummc.edu.my	University of Malaya	Kuala Lumpur		Nothing to declare
**Malaysia**	Wael Mohamed	waelmohamed@iium.edu.my	International Islamic University	Kuala Lumpur		Nothing to declare
**Malaysia**	Yi Wen Tay	tayyiwen96@um.edu.my	University of Malaya	Kuala Lumpur		Nothing to declare
**Malaysia**	Lim Kai-Shi	limkaishi612@gmail.com	University of Malaya	Kuala Lumpur		Nothing to declare
**Malaysia**	Azalea Tenerife Pajo	azaleapajo@gmail.com	University of Malaya	Kuala Lumpur		Nothing to declare
**Malaysia**	Chia Yuen Kang	cyk1118@gmail.com	Hospital Queen Elizabeth	Kota Kinabalu		Nothing to declare
**Malaysia**	Joshua Ooi Chin Ern	joshuaooichinern@gmail.com	Queen Elizabeth Hospital	Kota Kinabalu		Nothing to declare
**Malaysia**	Khairul Azmi Ibrahim	khairulazmi73@yahoo.com	HOSPITAL SULTANAH NUR ZAHIRAH KUALA TERENGGANU	KUALA TERENGGANU		Nothing to declare
**Malaysia**	Ahmad Shahir Bin Mawardi	sha_ray@yahoo.com	Hospita Kuala Lumpur	Kuala Lumpur		Nothing to declare
**Malaysia**	Lim Thien Thien	thienthienlim@gmail.com	Island Hospital	Penang		Nothing to declare
**Malaysia**	Tzi Shin Toh	tzishintoh@gmail.com	University of Malaya	Kuala Lumpur		Nothing to declare
**Mexico**	Daniel Martinez-Ramirez	daniel.martinez-ramirez@tec.mx	Tecnologico de Monterrey	Monterrey		Nothing to declare
**Mexico**	Paula Reyes-Pérez	prreyesperez@liigh.unam.mx	Universidad Nacional Autónoma de México	Santiago de Querétaro		Global Parkinson's Genetics Program
**Mexico**	Alejandra Medina	amedina@liigh.unam.mx	Universidad Nacional Autónoma de México	Santiago de Querétaro		Nothing to declare

	Rivera					
**Mexico**	Nancy Monroy Jaramillo	nancy.monroy@innn.edu.mx	Instituto Nacional de Neurología y Neurocirugía	Mexico City		Nothing to declare
**Mexico**	Nadia Alejandra Gandarilla Martinez	ngandarilla.neurologia@gmail.com	Centro Neurológico del Centro Médico ABC, Campus Santa Fe	Mexico City		Nothing to declare
**Mexico**	Ingrid Estrada-Bellmann	ingridestmann@hotmail.com	UNIVERSITY HOSPITAL “DR JOSE E GONZALEZ”	Monterrey		Nothing to declare
**Mexico**	Araliz Puente	aralizp@gmail.com	Hospital Ángeles Puebla	Puebla		Nothing to declare
**Mexico**	Ana Paula Angulo Arrieta	ana.anguloar@anahuac.mx	Hospital Ángeles Puebla, Universidad Anáhuac Puebla	Puebla		Nothing to declare
**Mexico**	Eugenia Morelos Figaredo	neuro.emf@gmail.com	ISSSTE Morelia	Morelia		Nothing to declare
**Mexico**	Karla Salinas Barboza	saurka24@gmail.com	HOSPITAL GENERAL DE MEXICO	Mexico City		Nothing to declare
**Mexico**	Dante Bernardo Oropeza Canto	danteoropeza2@hotmail.com	Hospital Angeles de Puebla	Puebla		Nothing to declare
**Mexico**	Mayela Rodríguez-Violante	mrodriguez@innn.edu.mx	Instituto Nacional de Neurología y Neurocirugía	Mexico City		Nothing to declare
**Mexico**	Ana Jimena Hernández-Medrano	anajimenahdz@gmail.com	Instituto Nacional de Neurología y Neurocirugía Manuel Velasco Suárez	Mexico City		Nothing to declare
**Mexico**	Amin Cervantes-Arriaga	acervantes@innn.edu.mx	Instituto Nacional de Neurología y Neurocirugía	Mexico City		Nothing to declare

**Mexico**	Edith Janeth Gaspar Martínez	edith.gaspar@protonmail.com	Universidad Nacional Autónoma de México	Santiago de Querétaro		Nothing to declare
**Mexico**	Alejandra E-Ruiz-Contreras	aleruiz@unam.mx	Universidad Nacional Autonoma de Mexico	Mexico City		Nothing to declare
**Mexico**	Alejandra Lázaro-Figueroa	lazfigal@gmail.com	National Autonomous University of Mexico	Mexico City		Nothing to declare
**Mongolia**	Bayasgalan Tserensodnom	bayasgalan.ts@mnums.edu.mn	Mongolian National University of Medical Sciences	Ulaanbaatar		Nothing to declare
**Mongolia**	Khosbayar Tulgaa	khosbayar.t@mnums.edu.mn	Mongolian National University of Medical Sciences	Ulaanbaatar		Nothing to declare
**Mongolia**	Oyujin Ulziibaatar	ou252@nyu.edu	Mongolian National University of Medical Sciences	Ulaanbaatar		Nothing to declare
**Morocco**	Ahmed Bouhouche	a.bouhouche@um5r.ac.ma	Specialties Hospital, CHU Ibn Sina	Rabat		Nothing to declare
**Morocco**	Mossafa Hossain	hossein.mossafa@gmail.com	Clinique OCEANIC	CASABLANCA		Nothing to declare
**Nepal**	Rajeev Ojha	rajeevnet@hotmail.com	Tribhuvan University	Kirtipur		Nothing to declare
**Netherlands**	Wilma Van De Berg	wdj.vandeberg@amsterdamumc.nl	Vanderbilt University Medical Center	Amsterdam		Nothing to declare
**Netherlands**	Bas Bleom	bas.bloem@radboudumc.nl	Radboud University	Nijmegen		Nothing to declare
**Netherlands**	Bart Van De Warrenburg	bart.vandewarrenburg@radboudumc.nl	Radboud University Medical Center	Nijmegen		Nothing to declare
**Netherlands**	Lisette Charbonnier	l.charbonnier@lumc.nl	Brain Research and Innovation Center	Amsterdam		Nothing to declare
**New Zealand**	Tim J. Anderson	tim.anderson@otago.ac.nz	University of Otago	Dunedin		Heath Research Council of New Zealand; Ministry of Business Innovation and Employment (New Zealand), Neurological

						Foundation of New Zealand
**New Zealand**	Toni L. Pitcher	toni.pitcher@nzbri.org	University of Otago	Dunedin		Funding - Health Research Council of New Zealand
**New Zealand**	Daniel Jeremy Myall	daniel.myall@nzbri.org	New Zealand Brain Research Institution	Christchurch		Nothing to declare
**New Zealand**	John C. Dalrymple-Alford	john.dalrymple-alford@canterbury.ac.nz	University of Canterbury	Christchurch		Nothing to declare
**Nigeria**	Arinola Sanyaolu	asanyaolu@unilag.edu.ng	University of Lagos	Lagos		Nothing to declare
**Nigeria**	Njideka Okubadejo	nokubadejo@unilag.edu.ng	University of Lagos	Lagos		Michael J Fox Foundation; Tertiary Education Trust Fund (TETFUND) National Research Fund
**Nigeria**	Lara Ojo	oluojo@unilag.edu.ng	University of Lagos	Lagos		Nothing to declare
**Nigeria**	Oluwadamilola Ojo	oluojo@unilag.edu.ng	College of Medicine of the University of Lagos	Lagos		Nothing to declare
**Nigeria**	Simon Izuchukwu Ozomma	simonozomma@yahoo.com	University of Calabar Teaching Hospital	Calabar		Nothing to declare
**Nigeria**	Kolawole Wahab	wahab.kw@unilorin.edu.ng	University of Ilorin	Ilorin		Nothing to declare
**Nigeria**	Oladunni Abiodun	oladunnidare@yahoo.com	General Hospital	Lagos		Nothing to declare
**Nigeria**	Sani Abubakar	saabubakar@abu.edu.ng	Ahmadu Bello University	Kaduna State		Nothing to declare
**Nigeria**	Fatimah Abdulai	fatimaabdulai32@yahoo.com	University of Abuja Teaching Hospital	Gwagwalada		Nothing to declare
**Nigeria**	Charles Achoru	charlesachoru82@gmail.com	Jos University Teaching Hospital	Jos		Nothing to declare
**Nigeria**	Osigwe Agabi	oagabi@unilag.edu.ng	College of Medicine, University of Lagos	Lagos		Nothing to declare

**Nigeria**	Uchechi Agulanna	ucheagulanna@gmail.com	Lagos University Teaching Hospital	Lagos		Nothing to declare
**Nigeria**	Rufus Akinyemi	roakinyemi@com.ui.edu.ng	Neuroscience and Ageing Research Unit, Institute for Advanced Medical Research and Training, College of Medicine, University of Ibadan	Ibadan		Nothing to declare
**Nigeria**	Wemimo Alaofin	awalaofin@gmail.com	University of Ilorin	Ilorin		Nothing to declare
**Nigeria**	Ifeyinwa Ani-Osheku	i.ani-osheku@nileuniversity.edu.ng	Asokoro District Hospital	Abuja		Nothing to declare
**Nigeria**	Roosevelt Anyanwu	ranyanwu@unilag.edu.ng	College of Medicine, University of Lagos	Lagos		Nothing to declare
**Nigeria**	Ohwotemu Arigbodi	ohwotemu@delsu.edu.ng	Delta State University	Abraka		Nothing to declare
**Nigeria**	Abiodun Bello	abiodunhbello@gmail.com	University of Ilorin Teaching Hospital	Ilorin		Nothing to declare
**Nigeria**	Cyril Erameh	cyrilerameh@isth.com.ng	Irrua Specialist Teaching Hospital	Ilorin		Nothing to declare
**Nigeria**	Daniel Ezuduemoih	danpasezudu@gmail.com	Lagos University Teaching Hospital	Lagos		Nothing to declare
**Nigeria**	Temitope Farombi	temitopefarombi@gmail.com	University College Hospital	Ibadan		Nothing to declare
**Nigeria**	Abdullahi Ibrahim	dribrahimbichi@gmail.com	Federal University of Health Sciences Teaching Hospital	Azare		Nothing to declare
**Nigeria**	Ahmed Idowu	melodocious@yahoo.com	Obafemi Awolowo University Teaching Hospitals Complex	Ile-Ife		Nothing to declare
**Nigeria**	Erica Ikwenu	ikwenuerica@gmail.com	Lagos University Teaching Hospital	Lagos		Nothing to declare
**Nigeria**	Frank Imarhiagbe	frank.imarhiagbe@uniben.edu	University of Benin	Benin City		Nothing to declare
**Nigeria**	Ismaila Ishola	iishola@unilag.edu.ng	College of Medicine, University of Lagos	Lagos		Nothing to declare
**Nigeria**	Emmanuel Iwuozo	emmanuel.iwuozo@npmcn.edu.ng	Benue State University	Makurdi		Nothing to declare
**Nigeria**	Morenikeji	morenikeji.komolaf	Obafemi Awolowo University	Ile-Ife		Nothing to declare

	Komolafe	e@oauife.edu.ng				
**Nigeria**	Alero Nnama	ibifurolove@gmail.com	University of Port Harcourt Teaching Hospital	Port Harcourt		Nothing to declare
**Nigeria**	Paul Nwani	po.nwani@unizik.edu.ng	Nnamdi Azikiwe University Teaching Hospital	Nnewi		Nothing to declare
**Nigeria**	Franscisca Nwaokorie	fnwaokorie@unilag.edu.ng	College of Medicine, University of Lagos	Lagos		Nothing to declare
**Nigeria**	Ernest Nwazor	ernestnwazor@yahoo.com	Rivers State University Teaching Hospital	Port Harcourt		Nothing to declare
**Nigeria**	Yakub Nyandaiti	nyandaiti@unimaid.edu.ng	University of Maiduguri Teaching Hospital	Maiduguri		Nothing to declare
**Nigeria**	Yahaya Obiabo	obiabo.yahaya@fuhso.edu.ng	Federal University of Health Sciences	Otukpo		Nothing to declare
**Nigeria**	Nkechi Obianozie	nkechiobian@gmail.com	University of Abuja Teaching Hospital	Gwagwalada		Nothing to declare
**Nigeria**	Olanike Odeniyi	nikeodeniyi@lagosstate.gov.ng	General Hospital	Lagos		Nothing to declare
**Nigeria**	Francis Odiase	francisodiase2000@hotmail.com	University of Benin	Benin City		Nothing to declare
**Nigeria**	Ewere Marie Ogbimi	eweremarie@gmail.com	Delta State University	Abraka		Nothing to declare
**Nigeria**	Adebimpe Ogunmodede	bimpefunmilola@yahoo.com	Federal Medical Center	Owo		Nothing to declare
**Nigeria**	Francis Ojini	fojini@unilag.edu.ng	University of Lagos	Lagos		Nothing to declare
**Nigeria**	Rashidat Olanigan	rolanigan@hotmail.com	Lagos State University Teaching Hospital	Ikeja		Nothing to declare
**Nigeria**	Adedunni Olusanya	dedunolusanya@gmail.com	College of Medicine, University of Lagos & R-Jolad Hospital	Lagos		Nothing to declare
**Nigeria**	Chiamaka Okereke	chiamakaokereke@unth.edu.ng	University of Nigeria Teaching Hospital	Ituku-Ozalla		Nothing to declare
**Nigeria**	Gerald Onwuegbuzie	gerald.onwuegbuzie@uniabuja.edu.ng	University of Abuja	Abuja		Nothing to declare

**Nigeria**	Godwin Osaigbovo	osaigbovog@unijos.edu.ng	Jos University Teaching Hospital	Jos		Nothing to declare
**Nigeria**	Nosakhare Osemwegie	nosakhare.osemwegie@uniport.edu.ng	University of Port Harcourt	Port Harcourt		Nothing to declare
**Nigeria**	Olajumoke Oshinaike	olufemi.oshinaike@lasucom.edu.ng	Lagos State University College of Medicine	Ikeja		Nothing to declare
**Nigeria**	Folajimi Otubogun	folabogun@gmail.com	Federal Medical Center	Lagos		Nothing to declare
**Nigeria**	Lukman Owolabi	olfemi.med@buk.edu.ng	Bayero University Kano	Kano		Nothing to declare
**Nigeria**	Raymond Owolabi	owolabiray@yahoo.com	Federal Medical Center	Owo		Nothing to declare
**Nigeria**	Shyngle Oyakhire	soyakhire@nationalhospital.gov.ng	National Hospital	Abuja		Nothing to declare
**Nigeria**	Fadimatu Sa'Ad	falmatasaad16@gmail.com	Federal Teaching Hospital	Gombe		Nothing to declare
**Nigeria**	Sarah Samuel	samuelsarah1012@gmail.com	University of Maiduguri Teaching Hospital	Maiduguri		Nothing to declare
**Nigeria**	Funmilola Taiwo	loleneuro@gmail.com	University College Hospital	Ibadan		Nothing to declare
**Nigeria**	Yusuf Zubair	dryusufzubair@gmail.com	National Hospital	Abuja		Nothing to declare
**Norway**	Lasse Pihlstrøm	lasse.pihlstrom@medisin.uio.no	Oslo University Hospital	Oslo		Southeastern Regional Health Authority, Norway and Michael J. Fox Foundation
**Norway**	Manuela Tan	manuela.tan@medisin.uio.no	Oslo University Hospital	Oslo		Southeastern Norway Regional Health Authority Norway and Michael J. Fox Foundation
**Norway**	Ingeborg Haugesag Lie	i.h.lie@studmed.uio.no	Oslo University Hospital	Oslo		Nothing to declare
**Norway**	Jodi Maple-Grødem	jodi.maple@uis.no	Stavanger University Hospital	Stavanger		Nothing to declare

**Norway**	Solveig E J Dalbro	soejac@ous-hf.no	Oslo University Hospital	Oslo		Nothing to declare
**Norway**	Ellen Hoven Maurtveten	emaurtve@ous-hf.no	Oslo University Hospital	Oslo		Nothing to declare
**Pakistan**	Shoaib Ur-Rehman	dr.shoaib@ustb.edu.pk	University of Science and Technology Bannu	Bannu		Nothing to declare
**Pakistan**	Mohamed Nour	man.nour22@gmail.com	Razi Hospital	Rawalpindi		Nothing to declare
**Peru**	Mario Cornejo-Olivas	mario.cornejo.o@incngen.org.pe	Universidad Cientifica del Sur	Lima		Michael J. Fox Foundation for Parkinson's Research and Aligning Science Across Parkinson's Initiative
**Philippines**	Maria Leila Doquenia	marialeiladoquenia@gmail.com	Metropolitan Medical Center	Manila		Nothing to declare
**Philippines**	Raymond Rosales	rlrosales@ust.edu.ph	Metropolitan Medical Center	Manila		Nothing to declare
**Philippines**	Gerard Saranza	gerardsaranza@gmail.com	Chong Hua Hospital	Cebu		Nothing to declare
**Poland**	Agata Gajos	agata.gajos@umed.lodz.pl	Medical University of Lodz	Lodz		Nothing to declare
**Russia**	Elena Iakovenko	helenaabracham@gmail.com	Research Center of Neurology	Moscow		Nothing to declare
**Russia**	Anna Gareeva	annagareeva@yandex.ru	Ufa Federal Research Center	Ufa		Nothing to declare
**Russia**	Gulnara Akhmadeeva	nevrolog.ufa@gmail.com	Ufa Scientific Center	Ufa		Nothing to declare
**Russia**	Irina Gilyazova	gilyasova_irina@mail.ru	Russian Academy of Sciences / Bashkir State Medical University	Ufa		Nothing to declare
**Saudi Arabia**	Bashayer Al Mubarak	bal-mubarak@kfshrc.edu.sa	King Faisal Specialist Hospital and Research Center	Riyadh		Nothing to declare
**Saudi Arabia**	Muhammad Umair	umairmu@ngha.med.sa	King Abdullah International Medical Research Center	Jeddah		Nothing to declare
**Serbia**	Nataša Dragašević	ntdragasevic@gmail.com	Neurology Clinic, University Clinical Center of Serbia	Belgrade		Nothing to declare

	Mišković					
**Serbia**	Andona Milovanović	andona8@gmail.com	Neurology Clinic, University Clinical Center of Serbia	Belgrade		Nothing to declare
**Singapore**	Eng-King Tan	tan.eng.king@singhealth.com.sg	National Neuroscience Institute	Singapore		Singapore National Medical Research Council (MOH-OFLCG-000207)
**Singapore**	Jia Nee Foo	jianee.foo@ntu.edu.sg	Nanyang Technological University	Singapore		Singapore National Medical Research Council (MOH-000559)
**Singapore**	Elaine Chew	elaine.chew@ntu.edu.sg	Nanyang Technological University	Singapore		Nothing to declare
**Slovenia**	Vesna Van Midden	vesna.van.midden@gmail.com	Ljubljana University Medical Centre	Ljubljana		Nothing to declare
**South Africa**	Ferzana Amod	ferzanaamo@ialch.co.za	University of KwaZulu-Natal	Durban		Nothing to declare
**South Africa**	Jonathan Carr	jcarr@sun.ac.za	University of Stellenbosch	Stellenbosch		Nothing to declare
**South Africa**	Soraya Bardien	sbardien@sun.ac.za	Stellenbosch University	Stellenbosch		National Research Foundation of South Africa[Grant Number 129249]
**South Africa**	Nikita Pillay	nikitasipi40@gmail.com	University of the Western Cape	Bellville		Nothing to declare
**South Africa**	Kathryn Step	kathrynstep@sun.ac.za	Stellenbosch University	Cape Town		Nothing to declare
**South Africa**	Riaan Van Coller	rvcoller@gmail.com	University of Pretoria	Pretoria		Nothing to declare
**South Korea**	Beomseok Jeon	brain@snu.ac.kr	Seoul National University Hospital	Seoul		Nothing to declare
**South Korea**	Yun Joong Kim	yunjkim@yuhs.ac	Yongin Severance Hospital	Seoul		Nothing to declare
**South Korea**	Jung Hwan Shin	neosjh2009@gmail.com	Seoul National University	Seoul		Nothing to declare
**South Korea**	Joowon Jang	jereiard@naver.com	Seoul National University	Seoul		Nothing to declare

**South Korea**	Jee-Young Lee	wieber04@snu.ac.kr	SMG-SNU Boramae Medical Center, College of Medicine Seoul National University	Seoul		Dr. Lee has received a research grant of National Research Foundation (NRF) funded by the Korean Government (MSIT), a multidisciplinary research grant-in-aid and a focused research grant-in-aid from the Seoul Metropolitan Government Seoul National University (SMG-SNU) Boramae Medical Center, and a research grant from Gemvax, a speaker honorarium from SK chemicals and Bial, a travel support from Ono, and a scientific advisory board of Regeners, Inc.
**Spain**	Esther Cubo	ecubo@ubu.es	Hospital Universitario Burgos	Burgos		Nothing to declare
**Spain**	Ignacio Alvarez	ignacio.alvafer@gmail.com	University Hospital Mutua Terrassa	Barcelona		Nothing to declare
**Spain**	Janet Hoenicka	janet.hoenicka@sjd.es	Institut de Recerca Sant Joan de Deu	Barcelona		Fondo de Investigación Sanitaria, Instituto Salud Carlos III, Grant PI019/00126
**Spain**	Katrin Beyer	kbeyer@igtp.cat	Research Institute Germans Trias i Pujol	Barcelona		Nothing to declare
**Spain**	Maria Teresa Periñan	t.perinantocino@qmul.ac.uk	Instituto de Biomedicina de Sevilla	Seville		Nothing to declare
**Spain**	Pau Pastor	pastorpau@gmail.com	University Hospital Germans Trias i Pujol	Barcelona		Nothing to declare
**Spain**	Ruben Fernandez-Santiago	ruben.fernandez.santiago@gmail.com	Hospital Clínic de Barcelona	Barcelona		Nothing to declare
**Spain**	Pilar Gómez Garre	mgomez-ibis@us.es	Instituto de Biomedicina de Sevilla	Seville		Nothing to declare
**Spain**	Pablo Mir	pmir@us.es	Instituto de Biomedicina de Sevilla	Seville		Nothing to declare
**Spain**	Mario	ezquerra@recerca.c	FCRB-IDIBAPS	Barcelona		Nothing to declare

	Ezquerra	linic.cat				
**Spain**	Celia Painous Marti	painous@clinic.cat	Hospital Clinic Barcelona	Barcelona		Nothing to declare
**Spain**	Lola J. Díaz-Feliz	lolaj.diaz@estudiante.uam.es	Fernando Pessoa University, San Roque Hospital	Las Palmas de Gran Canaria		Nothing to declare
**Spain**	José Matías Arbelo González	jose.arbelo@hospitalessanroque.com	Hospital Universitario San Roque Las Palmas/ Universidad Fernando Pessoa Canarias (UFPC)	Las Palmas de Gran Canaria		Nothing to declare
**Spain**	Juan Carlos Martínez Castrillo	jcmcastrillo@gmail.com	Hospital Ramón y Cajal	Madrid		Nothing to declare
**Spain**	Marina Mata	mmataal@yahoo.es	Hospital Universitario Infanta Sofia	Madrid		Nothing to declare
**Spain**	Oriol De Fabregues	odefabregues@gmail.com	Hospital Universitari Vall d'Hebron	Barcelona		Nothing to declare
**Spain**	Lydia Vela-Desojo	lvela61@gmail.com	Hospital Universitario Fundación Alcorcón	Madrid		Nothing to declare
**Spain**	Manuel Menendez Gonzalez	menendezgmanuel@uniovi.es	Hospital Universitario Central de Asturias	Oviedo		Nothing to declare
**Spain**	Yaroslau Compta	ycompta@clinic.cat	IDIBAPS / Hospital Clinic	Barcelona		Nothing to declare
**Spain**	Alicia Garrido	agarridop@clinic.cat	IDIBAPS-FCRB. Hospital Clinic Barcelona	Barcelona		Nothing to declare
**Spain**	Maria J Marti	mjmarti@clinic.cat	Hospital Clinic de Barcelona. Institut d'Investigacio Biomedica August Pi i Sunyer (IDIBAPS)	Barcelona		Nothing to declare
**Spain**	Almudena Sánchez-Gómez	alsanchez@clinic.cat	Hospital Clinic of Barcelona	Barcelona		Nothing to declare
**Spain**	Alexia T. Sánchez Reyes	investigacionUTM@ufpcanarias.es	Universidad Fernando Pessoa Canarias	Las Palmas de Gran Canaria		Nothing to declare
**Spain**	Laia Muñoz	lmunozl@santpau.c	IR Sant Pau	Barcelona		Nothing to declare

	Llahuna	at				
**Spain**	Joaquim Aumatell Escabies	jaumatelle@santpau.cat	IR SANT PAU	Barcelona		Nothing to declare
**Spain**	Javier Pagonabarraga Mora	jpagonabarraga@santpau.cat	IR SANT PAU	Barcelona		Nothing to declare
**Spain**	Ignacio Illán Gala	iillan@santpau.cat	IR SANT PAU	Barcelona		Nothing to declare
**Spain**	Esteban Muñoz	jemunoz@clinic.cat	Hospital Clínic de Barcelona	Barcelona		Nothing to declare
**Spain**	Manuela San Eufrasio Martínez	msaneufrasio-ibis@us.es	Instituto de Biomedicina de Sevilla	Sevilla		Nothing to declare
**Spain**	Laura Muñoz Delgado	lmunoz-ibis@us.es	Instituto de Biomedicina de Sevilla	Sevilla		Nothing to declare
**Spain**	Rafael Díaz Belloso	rdbelloso-ibis@us.es	Instituto de Biomedicina de Sevilla	Sevilla		Nothing to declare
**Spain**	Sergio García Díaz	sgarcia5@us.es	Instituto de Biomedicina de Sevilla	Sevilla		Nothing to declare
**Spain**	Marta Bonilla Toribio	mbonilla-ibis@us.es	Instituto de Biomedicina de Sevilla	Sevilla		Nothing to declare
**Spain**	Dolores Buiza Rueda	dbuiza-ibis@us.es	Instituto de Biomedicina de Sevilla	Sevilla		Nothing to declare
**Spain**	Antonio Cristobal Luque Ambrosiani	acluque-ibis@us.es	Instituto de Biomedicina de Sevilla	Sevilla		Nothing to declare
**Spain**	Silvia Jesus Maestre	smaestre-ibis@us.es	Instituto de Biomedicina de Sevilla	Sevilla		Nothing to declare
**Spain**	Daniel Macías	dmacias-ibis@us.es	Instituto de Biomedicina de Sevilla	Sevilla		Nothing to declare

	García					
**Spain**	Elena Ojeda Lepe	eojeda-ibis@us.es	Instituto de Biomedicina de Sevilla	Sevilla		Nothing to declare
**Spain**	Rocío Pineda Sánchez	rpineda-ibis@us.es	Instituto de Biomedicina de Sevilla	Sevilla		Nothing to declare
**Spain**	Ana Castellano Guerrero	acastellano-ibis@us.es	Instituto de Biomedicina de Sevilla	Sevilla		Nothing to declare
**Spain**	Astrid Daniela Adarmes Gómez	adarmes-ibis@us.es	Instituto de Biomedicina de Sevilla	Sevilla		Nothing to declare
**Spain**	Cristina Pérez Calvo	cperez6-ibis@us.es	Instituto de Biomedicina de Sevilla	Seville		Nothing to declare
**Spain**	Alejandro Salguero Oviedo	asalguero-ibis@us.es	Instituto de Biomedicina de Sevilla	Sevilla		Nothing to declare
**Spain**	Lorena Clavijo Jiménez	lclavijo-ibis@us.es	Instituto de Biomedicina de Sevilla	Sevilla		Nothing to declare
**Sudan**	Sarah El-Sadig	Sarah.misbah@uofk.edu	Faculty of medicine university of Khartoum	Khartoum		Nothing to declare
**Sweden**	Kajsa Brolin	kajsa.atterling_brolin@med.lu.se	Lund University	Lund		Nothing to declare
**Sweden**	Per Svenningsson	per.svenningsson@ki.se	Karolinska Institute	Stockholm		Nothing to declare
**Sweden**	Maria Swanberg	maria.swanberg@med.lu.se	Lund University	Lund		Nothing to declare
**Switzerland**	Christiane Zweier	christiane.zweier@insel.ch	Inselspital Bern, University of Bern	Bern		Nothing to declare
**Switzerland**	Gerd Tinkhauser	gerd.tinkhauser@insel.ch	University Hospital Bern	Bern		Nothing to declare
**Switzerla**	Paul Krack	paul.krack@insel.c	Inselspital Bern, University of Bern	Bern		Nothing to declare

**nd**		h				
**Switzerland**	Deborah Bartholdi	Deborah.Bartholdi@Insel.ch	University Hospital Bern	Bern		Nothing to declare
**Taiwan**	Chin-Hsien Lin	chlin@ntu.edu.tw	National Taiwan University Hospital	Taipei City		Nothing to declare
**Taiwan**	Hsiu-Chuan Wu	serenahcwu@gmail.com	Chang Gung Memorial Hospital	Taoyuan City		Nothing to declare
**Taiwan**	Pin-Jui Kung	pinjuikung@gmail.com	National Taiwan University	Taipei City		Global Parkinson's Genetics Program
**Taiwan**	Ruey-Meei Wu	robinwu@ntu.edu.tw	National Taiwan University Hospital	Taipei City		Minister of Science and Technology, Taiwan Government; National Taiwan University; Parkinson foundation (USA); Michael J. Fox Foundation
**Taiwan**	Yihru Wu	yihruwu@adm.cgmh.org.tw	Chang Gung Memorial Hospital	Taoyuan City		Nothing to declare
**Taiwan**	Pin-Shiuan, Chen	calice2657@gmail.com	National Taiwan University Hospital	Taipei		Nothing to declare
**Tajikistan**	Ganieva Manizha	ganieva.manizha.79@mail.ru	Avicenna Tajik State Medical University	Dushanbe		Nothing to declare
**Tajikistan**	Maksudjon Isrofilov	dr.maks-55@mail.ru	Avicenna Tajik State Medical University	Dushanbe		Nothing to declare
**Tunisia**	Rim Amouri	rim.amouri@rns.tn	Mongi Ben Hmida National Institute of Neurolog	Tunis		Nothing to declare
**Tunisia**	Samia Ben Sassi	dr.samiabensassi@gmail.com	Mongi Ben Hmida National Institute of Neurology	Tunis		Nothing to declare
**Tunisia**	Chokri Mhiri	mhiri.chokri@gmail.com	Habib Bourguiba University Hospital	Sfax		Nothing to declare
**Tunisia**	Nabli Fatnassi Fatma	fatmafatnassi@yahoo.fr	National institute Mongi Ben Hmida of Neurology	Tunis		Nothing to declare
**Tunisia**	Amine Rachdi	Dr.rachdi.amine@gmail.com	Mongi Ben Hamida institute of Neurology			Nothing to declare
**Tunisia**	Zakaria	zakariasaied@hotm	National Institute Mongi Ben Hamida of Neurology	Tunis		Nothing to declare

	Saied	ail.com				
**Tunisia**	Mouna Ben Djebara	mouna.bendjebara@gmail.com	Razi Hospital	Tunis		Nothing to declare
**Tunisia**	Rania Zouari	zouari.rania12@gmail.com	National institute of neurology mongi ben hmida	Tunis		Nothing to declare
**Turkey**	A. Nazlı Başak	nbasak@ku.edu.tr	Koç University	Istanbul		Kirac Foundation and Koc Univ.
**Turkey**	Özgür Öztop Çakmak	ooztop@ku.edu.tr	Koç University	Istanbul		Nothing to declare
**Turkey**	Sibel Ertan	fserta@istanbul.edu.tr	Koç University	Istanbul		Nothing to declare
**Turkey**	Rezzak Yilmaz	Rezzak.Yilmaz@ankara.edu.tr	University of Ankara	Ankara		RY received grants from Ankara University, and honoraria/consultancy fees from Abbvie, Gen, Ali Raif, Abdi Ibrahim, and advisory board contribution from Gen, and Abbvie.
**Turkey**	Binnur Çelik	binnurcelik@hotmail.com	University of Ankara	Ankara		Nothing to declare
**Turkey**	Gençer Genç	gencerg@gmail.com	Şişli Etfal Training and Research Hospital, University of Health Sciences, İstanbul, TR	Istanbul		Nothing to declare
**Turkey**	Muhittin Cenk Akbostancı	akbostan@medicine.ankara.edu.tr	Private Practice	Ankara		Nothing to declare
**Turkey**	Basar Bilgic	bilgicb@gmail.com	Istanbul University, Faculty of Medicine	Istanbul		Nothing to declare
**Turkey**	Bedia Samanci	bediasamanci@gmail.com	Istanbul University, Faculty of Medicine	Istanbul		Nothing to declare
**Turkey**	Murat Emre	muratemre56@gmail.com	Istanbul university	Istanbul		Nothing to declare
**Turkey**	Haşmet Hanağasi	hasmet@yahoo.com	ISTANBUL FACULTY OF MEDICINE	Istanbul		Nothing to declare
**Turkey**	Aysegul Gunduz	draysegulgunduz@yahoo.com	Istanbul University-Cerrahpasa, Cerrahpasa Faculty of Medicine	Istanbul		Nothing to declare

**Turkey**	Gulcin Benbir Senel	gulcin.benbir@iuc.edu.tr	Istanbul University-Cerrahpasa, Cerrahpasa Faculty of Medicine	Istanbul		Istanbul University-Cerrahpasa, Cerrahpasa Faculty of Medicine, Department of Neurology, Sleep and Disorders Unit, Istanbul, Turkiye
**UK**	Alastair Noyce	a.noyce@qmul.ac.uk	Queen Mary University of London	London		Prof. Noyce reports grants from Parkinson's UK, Barts Charity, Cure Parkinson's, NIHR, Innovate UK, Virginia Keiley benefaction, Alchemab, Aligning Science Across Parkinson's and Michael J Fox Foundation. Consultancy and personal fees from Astra Zeneca, AbbVie, Profile, Roche, Biogen, UCB, Bial, Charco Neurotech, uMedeor and Britannia.
**UK**	Anette Schrag	A.schrag@ucl.ac.uk	University College London	London		NIHR UCL/H Biomedical Research Centre
**UK**	Anthony Schapira	a.schapira@ucl.ac.uk	University College London	London		ASAP
**UK**	Camille Carroll	camille.carroll@plymouth.ac.uk	University of Plymouth	Plymouth		C Carroll receives salary from University of Plymouth, University Hospitals Plymouth NHS Trust and National Institute of Health Research; she has received advisory, consulting, and/or lecture fees from AbbVie, Bial, Lundbeck, Global Kinetics, Britannia and Medscape, and research funding from Parkinson's UK, Edmond J Safra Foundation, National Institute of Health Research and Cure

						Parkinson’s
**UK**	Donald Grosset	donald.grosset@glasgow.ac.uk	University of Glasgow	Glasgow		Tracking Parkinson's (J-1101) is funded by Parkinson's UK
**UK**	Eleanor J. Stafford	e.stafford@ucl.ac.uk	University College London	London		Nothing to declare
**UK**	Henry Houlden	h.houlden@ucl.ac.uk	University College London	London		Nothing to declare
**UK**	Huw R Morris	h.morris@ucl.ac.uk	University College London	London		Dr Morris is employed by UCL. In the last 12 months he reports paid consultancy from Roche and Amylyx ; lecture fees/honoraria - BMJ, Kyowa Kirin, Movement Disorders Society. Research Grants from Parkinson’s UK, Cure Parkinson’s Trust, PSP Association, CBD Solutions, Drake Foundation, Medical Research Council, Michael J Fox Foundation. Dr Morris is a co-applicant on a patent application related to C9ORF72 - Method for diagnosing a neurodegenerative disease (PCT/GB2012/052140)
**UK**	John Hardy	j.hardy@ucl.ac.uk	University College London	London		Nothing to declare
**UK**	Kin Ying Mok	k.mok@ucl.ac.uk	Univeristy College London	London		Nothing to declare
**UK**	Mie Rizig	m.rizig@ucl.ac.uk	University College London	London		Nothing to declare
**UK**	Nicholas Wood	n.wood@ucl.ac.uk	University College London	London		ASAP- CRN

**UK**	Nigel Williams	williamsnm@cardiff.ac.uk	Cardiff University	Cardiff		Parkinson's UK
**UK**	Olaitan Okunoye	olaitan.okunoye.16@ucl.ac.uk	University College London	London		Nothing to declare
**UK**	Rauan Kaiyrzhanov	rauan.kaiyrzhanov.14@ucl.ac.uk	University College London	London		Nothing to declare
**UK**	Rimona Weil	r.weil@ucl.ac.uk	University College London	London		Nothing to declare
**UK**	Seth Love	seth.love@bristol.ac.uk	University of Bristol	Bristol		Medical Research Council (UK) MR/T018569/1
**UK**	Simona Jasaityte	simona.jasaityte@ucl.ac.uk	University College London	London		Nothing to declare
**UK**	Sumit Dey	s.dey@qmul.ac.uk	Queen Mary University of London	London		The Michael J. Fox Foundation for Parkinson's Research
**UK**	Spencer Finch	s.finch@qmul.ac.uk	Queen Mary University of London	London		Nothing to declare
**UK**	Valentina Escott-Price	escottpricev@cardiff.ac.uk	Cardiff University	Cardiff		Nothing to declare
**UK**	Hamin Lee	hamin.lee@ucl.ac.uk	St George's, University of London	London		Nothing to declare
**UK**	Roger Barker	rab46@cam.ac.uk	University of Cambridge	Cambridge		Nothing to declare
**UK**	Mina Ryten	mina.ryten@ucl.ac.uk	University College London	London		Nothing to declare
**UK**	Michele Hu	michele.hu@ndcn.ox.ac.uk	University of Oxford	Oxford		Nothing to declare
**UK**	Laura Parkkinen	laura.parkkinen@ndcn.ox.ac.uk	University of Oxford	Oxford		Nothing to declare
**UK**	Kailash Bhatia	k.bhatia@ucl.ac.uk	University College London	London		Nothing to declare
**UK**	Richard Walker	richard.walker@nhct.nhs.uk	Northumbria Healthcare at NHS Foundation Trust	Newcastle upon Tyne		Nothing to declare

**UK**	Steve Gentleman	s.gentleman@imperial.ac.uk	Imperial College London	London		Nothing to declare
**UK**	Thomas Warner	t.warner@ucl.ac.uk	University College London	London		Nothing to declare
**UK**	David Burn	david.burn@newcastle.ac.uk	Newcastle University	Newcastle upon Tyne		Nothing to declare
**UK**	Christian Lambert	christian.lambert@ucl.ac.uk	Imperial College London	London		Nothing to declare
**UK**	Caroline Williams-Gray	chm27@cam.ac.uk	University of Cambridge	Cambridge		Nothing to declare
**UK**	Deborah Attuah	deborah.attuah@yld.io	YLD	London		Nothing to declare
**UK**	Raquel Real	r.real@ucl.ac.uk	University College London	London		Nothing to declare
**UK**	Yen Tai	yen.tai@imperial.ac.uk	Imperial College London	London		Nothing to declare
**UK**	Alexandra Zirra	a.zirra@qmul.ac.uk	Queen Mary University of London	London		Nothing to declare
**UK**	Christopher M Morris	c.m.morris@ncl.ac.uk	Newcastle University	Newcastle upon Tyne		Nothing to declare
**UK**	Matilda Lily Fenn	matildafenn1@gmail.com	University College London	London		Nothing to declare
**UK**	Andrew C Robinson	andrew.c.robinson@manchester.ac.uk	The University of Manchester	Manchester		Nothing to declare
**UK**	Lesley Yue Wu	lesleywu13@gmail.com	University College London	London		Nothing to declare
**UK**	Tessa Du Toit	tessa.toit.23@ucl.ac.uk	UCL	Londo		Nothing to declare
**UK**	Joshua Luc Isherwood Frost	joshua.frost@ucl.ac.uk	UCL Queen Square Institute of Neurology	London		Nothing to declare
**UK**	Federico	federico.roncaroli@	University of Manchester	Manchester		Nothing to declare

	Roncaroli	manchester.ac.uk				
**UK**	Ashvin Kuri	a.kuri2@nhs.net	Queen Mary University of London	London		Nothing to declare
**UK**	Sheena Waters	s.waters@qmul.ac.uk	Queen Mary University of London	London		Nothing to declare
**UK**	Laura Smith	l.j.smith@qmul.ac.uk	Queen Mary University of London	London		Nothing to declare
**UK**	Eduardo De Pablo-Fernández	e.depablofernandez@qmul.ac.uk	Queen Mary University of London	London		Nothing to declare
**UK**	Anisa Shahid	a.j.shahid@qmul.ac.uk	Queen Mary University of London	London		Nothing to declare
**UK**	Cristina Simonet	c.simonet@qmul.ac.uk	Queen Mary University of London	London		Nothing to declare
**UK**	Charlotte Dore	c.dore.17@ucl.ac.uk	University College London	London		Nothing to declare
**UK**	Oiher Serrano-Asensio	oiher.serrano.22@ucl.ac.uk	University College London	London		Nothing to declare
**UK**	Marco Toffoli	m.toffoli@ucl.ac.uk	University College London	London		Nothing to declare
**UK**	Riona Fumi	riona.fumi.20@ucl.ac.uk	University College London, Institute of Neurology	London		Nothing to declare
**UK**	Brook Huxford	b.f.r.huxford@qmul.ac.uk	Queen Mary University of London	London		Nothing to declare
**UK**	Harneek Chohan	h.chohan@qmul.ac.uk	Queen Mary University of London	London		Nothing to declare
**UK**	Sophie I Meyer	sophiemeyer165@gmail.com	Queen Mary University of London	London		Nothing to declare
**UK**	Laura Pérez-Carbonell	l.perezcarbonell@qmul.ac.uk	Queen Mary University London / Guy's and St Thomas' NHS Foundation Trust	London		Nothing to declare
**UK**	Solomiia Bandrivska	bandrivska.solomiia@gmail.com	University College London	London		Nothing to declare
**UK**	Saiesha	saieshadindayal@ro	University College London	London		Nothing to declare

	Dindayal	cketmail.com				
**UK**	Charlotte Andrews	c.andrews@qmul.ac.uk	Queen Mary University of London	London		Nothing to declare
**UK**	Emily Navarro Jones	emily.navarro-jones.23@ucl.ac.uk	Queen Mary University of London	London		Nothing to declare
**USA**	Lara M. Lange	lara.lange@nih.gov	Laboratory of Neurogenetics, National Institute on Aging/Institute of Neurogenetics, University of Luebeck	Bethesda	Maryland	Nothing to declare
**USA**	Alejandro Martínez-Carrasco	maal@broadinstitute.org	Broad Institute of MIT and Harvard	Cambridge	Massachusetts	Global Parkinson's Genetics Program
**USA**	Angel Vinuela		University of Puerto Rico	San Juan		Nothing to declare
**USA**	Alyssa O'Grady	aogrady@michaeljfox.org	The Michael J. Fox Foundation for Parkinson's Research	New York	New York	Nothing to declare
**USA**	Andrew B Singleton	andy@gp2.org	Global Parkinson’s Genetics Program (GP2)	Bethesda	Maryland	Michael J Fox Foundation for Parkinson's disease Research and Aligning Science Across Parkinson's Initiative
**USA**	Andrew K. Sobering	andrew.sobering@uga.edu	Augusta University / University of Georgia Medical Partnership	Augusta	Georgia	Nothing to declare
**USA**	Bernadette Siddiqi	bsiddiqi@michaeljfox.org	The Michael J. Fox Foundation for Parkinson's Research	New York	New York	Nothing to declare
**USA**	Bradford Casey	bcasey@michaeljfox.org	The Michael J. Fox Foundation for Parkinson's Research	New York	New York	Nothing to declare
**USA**	Brian Fiske	bfiske@michaeljfox.org	The Michael J. Fox Foundation for Parkinson's Research	New York	New York	Nothing to declare
**USA**	Cabell Jonas	cabell.jonas@kp.org	Mid-Atlantic Permanente Medical Group	Bethesda	Maryland	Nothing to declare

**USA**	Carlos Cruchaga	cruchagac@wustl.edu	Washington University	St. Louis	Missouri	National Institutes of Health (R01AG044546 (CC), P01AG003991(CC, JCM), RF1AG053303 (CC), RF1AG058501 (CC), U01AG058922 (CC), RF1AG074007 (YJS)), the Chuck Zuckerberg Initiative (CZI), the Michael J. Fox Foundation (LI, CC), and the Department of Defense (LI-W81XWH2010849). The recruitment and clinical characterization of research participants at Washington University were supported by NIH P30AG066444 (JCM), P01AG03991(JCM), and P01AG026276(JCM).
**USA**	Caroline B. Pantazis	cpantazis@aligningscience.com	Coalition for Aligning Science	Bethesda	Maryland	Nothing to declare
**USA**	Claire Wegel	cwegel@iu.edu	Indiana University	Bloomington	Indiana	Nothing to declare
**USA**	Cornelis Blauwendraat	cblauwendraat@aligningscience.com	Aligning Science Across Parkinson's (ASAP)	Bethesda	Maryland	Nothing to declare
**USA**	Dan Vitale	dan@datatecnica.com	Data Tecnica	Bethesda	Maryland	D.V.'s participation in this project was part of a competitive contract awarded to Data Tecnica International LLC by the National Institutes of Health to support open science research.
**USA**	Deborah Hall	deborah_a_hall@rush.edu	Rush University	Chicago	Illinois	Nothing to declare
**USA**	Dena	hernand@mail.nih.	National Institutes of Health	Bethesda	Marylan	Nothing to declare

	Hernandez	gov			d	
**USA**	Ekemini Riley	eriley@parkinsonsroadmap.org	Coalition for Aligning Science	Washington	Washington	Nothing to declare
**USA**	Faraz Faghri	faraz@datatecnica.com	Data Tecnica	Bethesda	Maryland	F.F.'s participation in this research was supported in part by the Intramural Research Program of the NIH, National Institute on Aging (NIA), National Institutes of Health, Department of Health and Human Services; project number ZO1 AG000535, as well as the National Institute of Neurological Disorders and Stroke. F.F.'s participation in this project was part of a competitive contract awarded to Data Tecnica International LLC by the National Institutes of Health to support open science research.
**USA**	Geidy E. Serrano	geidy.serrano@bannerhealth.com	Banner Sun Health Research Institute	Sun City	Arizona	Banner Sun Health Research Institute Brain and Body Donation Program of Sun City, Arizona for the provision of human biological materials. The Brain and Body Donation Program has been supported by the National Institute of Neurological Disorders and Stroke (U24 NS072026 National Brain and Tissue Resource for Parkinson's Disease and Related Disorders), the National Institute on Aging (P30 AG19610 and P30AG072980, Arizona

						Alzheimer's Disease Center), the Arizona Department of Health Services (contract 211002, Arizona Alzheimer's Research Center), the Arizona Biomedical Research Commission (contracts 4001, 0011, 05-901 and 1001 to the Arizona Parkinson's Disease Consortium) and the Michael J. Fox Foundation for Parkinson's Research
**USA**	Hampton Leonard	hampton@datatecnica.com	Data Tecnica	Bethesda	Maryland	H.L.L.'s participation in this project was part of a competitive contract awarded to Data Tecnica International LLC by the National Institutes of Health to support open science research.
**USA**	Hirotaka Iwaki	hirotaka@datatecnica.com	Data Tecnica	Washington	Washington	H.I.'s participation in this project was part of a competitive contract awarded to Data Tecnica International LLC by the National Institutes of Health to support open science research.
**USA**	Honglei Chen	chenho19@msu.edu	Michigan State University	East Lansing	Michigan	NIH/DoD/Parkinson Foundation/MSU Foundation/Gibby vs. Parky Foundation - No COI to disclose
**USA**	Ignacio F. Mata	matai@ccf.org	Cleveland Clinic	Cleveland	Ohio	Funding from MJFF and NIH
**USA**	Ignacio Juan Keller Sarmiento	ignacio.sarmiento@northwestern.edu	Northwestern University	Evanston	Illinois	Nothing to declare
**USA**	Jared Williamson	Jared.C.Williamson@kp.org	Kaiser Permanente	Oakland	California	Nothing to declare

**USA**	Jonggeol Jeff Kim	Jonggeol.Kim@bcm.edu	Baylor College of Medicine	Bethesda	Maryland	Nothing to declare
**USA**	Joseph Jankovic	pdcmdc@bcm.edu	Baylor College of Medicine	Houston	Texas	Nothing to declare
**USA**	Joshua Shulman	joshua.shulman@bcm.edu	Baylor College of Medicine / Texas Children's Hospital	Houston	Texas	Collection of samples and data at Baylor College of Medicine was supported by Huffington Foundation.
**USA**	J Solle	jsolle@michaeljfox.org	The Michael J. Fox Foundation for Parkinson's Research	New York	New York	Nothing to declare
**USA**	Kaileigh Murphy	kmurphy@michaeljfox.org	The Michael J. Fox Foundation for Parkinson's Research	New York	New York	Nothing to declare
**USA**	Kamalini Ghosh Galvelis	kghosh@parkinson.org	Parkinson's Foundation	Princeton	New Jersey	Nothing to declare
**USA**	Karen Nuytemans	knuytemans@med.miami.edu	University of Miami Miller School of Medicine	Miami	Florida	This work has been supported by the American Parkinson Disease Association and the Margaret Q. Landenberger Research Foundation.
**USA**	Karl Kieburtz	karl.kieburtz@chet.rochester.edu	Beth Israel Deaconess Medical Center	Boston	Massachusetts	Nothing to declare
**USA**	Katerina Markopoulou	amarkopoulou@northshore.org	North Shore University Health System	Chicago	Illinois	Nothing to declare
**USA**	Kenneth Marek	kmarek@indd.org	Institute for Neurodegenerative Disorders	New Haven	Connecticut	Consultant for Michael J Fox Foundation, GE Healthcare, Roche, UCB, BIAL, Denali, Takeda, , Cerapsir, UCB, Biohaven, Neuron23, Aprinoia, Astellas, Calico, Genentech, Invicro

**USA**	Kristin S. Levine	kristin@datatecnica.com	Data Tecnica	Washington	Washington	K.S.L.’s participation in this project was part of a competitive contract awarded to Data Tecnica International LLC by the National Institutes of Health to support open science research.
**USA**	Lana M. Chahine	lanachahine@pitt.edu	University of Pittsburgh	Pittsburgh	Pennsylvania	Nothing to declare
**USA**	Laura Ibanez	ibanezl@wustl.edu	Washington University	St. Louis	Missouri	Nothing to declare
**USA**	Laurel Screven	laurel@gp2.org	Global Parkinson’s Genetics Program (GP2)	Bethesda	Maryland	Nothing to declare
**USA**	Lauren Ruffrage	lruffrage@uabmc.edu	University of Alabama at Birmingham	Birmingham	Alabama	Nothing to declare
**USA**	Lisa Shulman	lshulman@som.umaryland.edu	University of Maryland	Baltimore	Maryland	Nothing to declare
**USA**	Luca Marsili	marsilla@ucmail.uc.edu	University of Cincinnati	Cincinnati	Ohio	Nothing to declare
**USA**	Maggie Kuhl	mkuhl@michaeljfox.org	The Michael J. Fox Foundation for Parkinson's Research	New York	New York	Nothing to declare
**USA**	Marissa Dean	mndean@uabmc.edu	University of Alabama at Birmingham	Birmingham	Alabama	Dr. Dean is an investigator in studies funded by Abbvie, Inc., Hoffmann-La Roche, CHDI Foundation, Inc., Annexon, Inc., Retrophin, Inc, Neurocrine Biosciences, UniQure Biopharma B.V., Praxis Precision Medicines, Neuraly, Inc., Michael J. Fox Foundation for Parkinson’s Research, and US Army Medical Research and Material Command (grant#W81XWH-18-1-0508). In addition, Dr. Dean receives support through the Huntington’s

						Disease Society of American Centers of Excellence program.
**USA**	Mary B Makarious	mary@datatecnica.com	Data Tecnica	Bethesda	Maryland	M.B.M.'s participation in this project was part of a competitive contract awarded to Data Tecnica International LLC by the National Institutes of Health to support open science research.
**USA**	Mathew Koretsky	mat@datatecnica.com	Data Tecnica	Bethesda	Maryland	M.K.'s participation in this project was part of a competitive contract awarded to Data Tecnica International LLC by the National Institutes of Health to support open science research.
**USA**	Megan J. Puckelwartz	m.puckelwartz@northwestern.edu	Northwestern University	Chicago	Illinois	Nothing to declare
**USA**	Mike A. Nalls	mike@datatecnica.com	Data Tecnica	Bethesda	Maryland	M.A.N.'s participation in this project was part of a competitive contract awarded to Data Tecnica International LLC by the National Institutes of Health to support open science research. M.A.N. also currently serves as an advisor for Clover Therapeutics and Neuron23 Inc.
**USA**	Naomi Louie	nlouie@michaeljfox.org	The Michael J. Fox Foundation for Parkinson's Research	New York	New York	Nothing to declare
**USA**	Niccolò Emanuele Mencacci	niccolo.mencacci@northwestern.edu	Northwestern University	Evanston	Illinois	Nothing to declare
**USA**	Roger Albin	ralbin@med.umich.edu	Universit of Michigan	Ann Arbor	Michigan	P50NS123067; Parkinson's Foundation

**USA**	Roy Alcalay	rna2104@cumc.columbia.edu	Columbia University	New York	New York	Dr. Alcalay is funded by the Micharl J. Fox Foundation and the Parkinson's Foundation. He received consultation fees from Avrobio, Caraway, GSK, Merck, Sanofi, Ono Therapeutics and Takeda
**USA**	Ruth Walker	ruth.walker@mssm.edu	James J. Peters Veterans Affairs Medical Center	New York	New York	Research funding from the Department of Veterans Affairs (CSRD Merit Award CX002342), consulted for Teladoc, Inc. and received an honorarium from New York University (NYU) Medical Center.
**USA**	Sara Bandres-Ciga	sara.bandresciga@nih.gov	National Institutes of Health	Bethesda	Maryland	Nothing to declare
**USA**	Sohini Chowdhury	schowdhury@michaeljfox.org	The Michael J. Fox Foundation for Parkinson's Research	New York	New York	Nothing to declare
**USA**	Sonya Dumanis	sdumanis@parkinsonsroadmap.org	Aligning Science Across Parkinson's	Washington	Washington	Nothing to declare
**USA**	Steven Lubbe	steven.lubbe@northwestern.edu	Northwestern University	Chicago	Illinois	Nothing to declare
**USA**	Tao Xie	txie@bsd.uchicago.edu	University of Chicago	Chicago	Illinois	Nothing to declare
**USA**	Tatiana Foroud	tforoud@iu.edu	Indiana University School of Medicine	Indianapolis	Indiana	The Michael J. Fox Foundation for Parkinson's Research

**USA**	Thomas Beach	thomas.beach@bannerhealth.com	Sun Health Research Institution	Sun City	Arizona	Banner Sun Health Research Institute Brain and Body Donation Program of Sun City, Arizona for the provision of human biological materials. The Brain and Body Donation Program has been supported by the National Institute of Neurological Disorders and Stroke (U24NS072026 National Brain and Tissue Resource for Parkinson's Disease and Related Disorders), the National Institute on Aging (P30 AG19610 and P30AG072980, Arizona Alzheimer's Disease Center), the Arizona Department of Health Services (contract 211002, Arizona Alzheimer's Research Center), the Arizona Biomedical Research Commission (contracts 4001, 0011, 05-901 and 1001 to the Arizona Parkinson's Disease Consortium) and the Michael J. Fox Foundation for Parkinson's Research
**USA**	Todd Sherer	tsherer@michaeljfox.org	The Michael J Fox Foundation for Parkinson's Research	New York	New York	Nothing to declare
**USA**	Dana Lewis	dlewis@parkinsonsroadmap.org	Aligning Science Across Parkinson's	Baltimore	Maryland	Nothing to declare
**USA**	Shreya Menon	shreya.menon@gladstone.ucsf.edu	Gladstone Institutes	San Francisco	California	Nothing to declare
**USA**	Melissa Nirenberg	melissa.nirenberg@gmail.com	Icahn School of Medicine at Mount Sinai	New York	New York	Nothing to declare

**USA**	Spencer Grant	spencer.grant@nih.gov	National Institutes of Health	Bethesda	Maryland	Nothing to declare
**USA**	Shannon Ballard	shannon@datatecnica.com	Data Tecnica	Bethesda	Maryland	S.B.’s participation in this project was part of a competitive contract awarded to Data Tecnica International LLC by the National Institutes of Health to support open science research.
**USA**	Chad Shaw	cashaw@bcm.edu	Baylor College of Medicine	Houston	Texas	Nothing to declare
**USA**	Sidra Aslam	Sidra.Aslam@bannerhealth.com	Banner Health	Phoenix	Arizona	Nothing to declare
**USA**	Devin Sharp	dsnyder@aligningscience.com	Aligning Science Across Parkinson's	Vancouver	British Columbia	Nothing to declare
**USA**	Rachel Saunders-Pullman	Rachel.Saunders-Pullman@mountsinai.org	Icahn School of Medicine at Mount Sinai	New York	New York	Nothing to declare
**USA**	Michiko Kimura Bruno	michikobruno@gmail.com	The Queen's Medical Center	Honolulu	Hawaii	Nothing to declare
**USA**	Matt Farrer	m.farrer@ufl.edu	University of Florida College of Medicine	Gainesville	Florida	Nothing to declare
**USA**	Haydeh Payami	haydehpayami@uabmc.edu	The University of Alabama at Birmingham Heersink School of Medicine	Birmingham	Alabama	Nothing to declare
**USA**	Ryan Pfingst	rpfingst@michaeljfox.org	The Michael J Fox Foundation	New York	New York	Nothing to declare
**USA**	James B Leverenz	leverej@ccf.org	Cleveland Clinic	Cleveland	Ohio	Nothing to declare
**USA**	Elizabeth Disbrow	elizabeth.disbrow@lsuhs.edu	LSU Health Shreveport	Shreveport	Louisiana	Nothing to declare
**USA**	Debi Brooks	dbrooks@michaeljfox.org	The Michael J Fox Foundation	New York	New York	Nothing to declare
**USA**	Randy Schekman	rschekman@parkinsonsroadmap.org	University of California, Berkeley	Berkeley	California	Nothing to declare
**USA**	Un Kang	un.kang@nyulango	NYU Grossman School of Medicine	New York	New	Nothing to declare

		ne.org			York	
**USA**	Zbigniew K. Wszolek	Wszolek.Zbigniew@mayo.edu	Mayo Clinic College of Medicine	Rochester	Minnesota	Nothing to declare
**USA**	Cyrus Zabetian	zabetian@uw.edu	VA Puget Sound Health Care System	Seattle	Washington	Nothing to declare
**USA**	Zach Chaney	zchaney@michaeljfox.org	The Michael J Fox Foundation	New York	New York	Nothing to declare
**USA**	Christine Swanson-Fischer	christine.swanson-fischer@nih.gov	National Institutes of Health	Rockville	Maryland	Nothing to declare
**USA**	Conor Hennessey	chennessey@michaeljfox.org	The Michael J Fox Foundation	New York	New York	Nothing to declare
**USA**	Cassandra Barrett	cbarrett@michaeljfox.org	The Michael J Fox Foundation	New York	New York	Nothing to declare
**USA**	Beate Ritz	britz@ucla.edu	University of California, Los Angeles	Los Angeles	California	Nothing to declare
**USA**	Bradley Boeve	bboeve@mayo.edu	Mayo Clinic	Rochester	Minnesota	Nothing to declare
**USA**	Ashley Rawls	ashley.rawls@neurology.ufl.edu	University of Florida College of Medicine	Gainesville	Florida	Nothing to declare
**USA**	Holly A. Shill	holly.shill@commonspirit.org	Barrow Neurological Institute	Phoenix	Arizona	Nothing to declare
**USA**	Erika Driver-Dunckley	driverdunckley.erika@mayo.edu	Mayo Clinic AZ	Scottsdale	Arizona	Nothing to declare
**USA**	Bruce A. Chase	bruce.chase@endeavorhealth.org	Endeavor Health (formerly NorthShore University Health System)	Skokie	Illinois	Nothing to declare
**USA**	Mahesh Padmanaban	mpadmanaban@uchicagomedicine.org	University of Chicago	Chicago	Illinois	Nothing to declare
**USA**	Thiago Peixoto Leal	PEIXOTT@ccf.org	Cleveland Clinic	Cleveland	Ohio	Thiago Peixoto Leal is supported by the National Institutes of Health (NIH) grant R01 1R01NS112499-01A1 and Veterans Affair Puget Sound

						Healthcare System grant 5I01ABX005978-2
**USA**	Owen A. Ross	ross.owen@mayo.edu	Mayo Clinic	Jacksonville	Florida	Nothing to declare
**USA**	Michael Rose	michael.rose@osumc.edu	The Ohio State University Medical Center	Columbus	Ohio	Nothing to declare
**USA**	Ariane Park	ariane.park@osumc.edu	The Ohio State University Medical Center	Columbus	Ohio	I have received grants or contracts from the Parkinson's Foundation, Michael J. Fox Foundation, Parkinson Study Group and honoraria for consultancy from Cerevel.
**USA**	Victoria Klee	victoria.klee@osumc.edu	The Ohio State University	Columbus	Ohio	Nothing to declare
**USA**	James C. Beck	jbeck@parkinson.org	Parkinson's Foundation	New York	New York	Nothing to declare
**USA**	Suzanne Judd	sejudd@uab.edu	UAB	Birmingham	Alabama	Nothing to declare
**USA**	Daniel Weintraub	daniel.weintraub@pennmedicine.upenn.edu	U. Pennsylvania	Philadelphia	Pennsylvania	Nothing to declare
**USA**	Vikas Kotagal	vikaskot@med.umich.edu	University of Michigan	Ann Arbor	Michigan	Nothing to declare
**USA**	Nicolaas I. Bohnen	nbohnen@med.umich.edu	University of Michigan	Ann Arbor	Michigan	Nothing to declare
**USA**	Prabesh Kanel	prabeshk@med.umich.edu	University of Michigan	Ann Arbor	Michigan	Nothing to declare
**USA**	Chatkaew Pongmala	pchatkae@med.umich.edu	University of Michigan	Ann Arbor	Michigan	Nothing to declare
**USA**	Erin Williams	erin.williams@vai.org	Van Andel Institute	Grand Rapids	Michigan	Nothing to declare
**USA**	Audrey Strongosky	strongosky.audrey@mayo.edu	Mayo Clinic Florida	Jacksonville	Florida	Nothing to declare
**USA**	Michael	michael.henderson	Van Andel Institute	Grand	Michiga	Nothing to declare

	Henderson	@vai.org		Rapids	n	
**USA**	Daniel C. Rohrer	dan.rohrer@vai.org	Van Andel Institute	Grand Rapids	Michigan	Nothing to declare
**USA**	Alexander Blanski	alex.blanski@vai.org	Van Andel Research Institute	Grand Rapids	Michigan	Nothing to declare
**USA**	Christina Missler	christina.missler@vai.org	Van Andel Institute	Grand Rapids	Michigan	Nothing to declare
**USA**	Alyssa Johansson	alyssa.johansson@vai.org	Van Andel Institute	Grand Rapids	Michigan	Nothing to declare
**USA**	Felipe Duarte-Zambrano	felipe.dz.101@gmail.com	Cleveland Clinic	Cleveland	Ohio	Nothing to declare
**USA**	Gist Croft	gcroft@nyscf.org	The New York Stem Cell Foundation	New York	New York	Nothing to declare
**USA**	Lisa Voltolina	lvoltolina@nyscf.org	New York Stem Cell Foundation Reseach Institute	New York	New York	Nothing to declare
**USA**	Whitley Aamodt	whitley.aamodt@pennmedicine.upenn.edu	University of Pennsylvania	Philadelphia	Pennsylvania	Nothing to declare
**USA**	Stewart A Factor	sfactor@emory.edu	Emory University	Atlanta	Georgia	Nothing to declare
**USA**	Alberto J. Espay	Alberto.espay@uc.edu	University of Cincinnati	Cincinnati	Ohio	Nothing to declare
**USA**	Nabila Dahodwala	nabila.dahodwala@gmail.com	University of Pennsyvlania	Philadelphia	Pennsylvania	Nothing to declare
**USA**	Chantale Branson	cbranson@msm.edu	Morehouse School of Medicine	Atlanta	Georgia	Nothing to declare
**USA**	Emily Hill	hill2e9@ucmail.uc.edu	University of Cincinnati	Cincinnati	Ohio	Nothing to declare
**USA**	Krutika Patel	krutika.patel.co@gmail.com	New York Stem Cell Foundation	Denver	Colorado	Nothing to declare
**USA**	Shyamal Mehta	mehta.shyamal@mayo.edu	Mayo Clinic, Arizona	Scottsdale	Arizona	Nothing to declare
**USA**	Emily Waldo	waldoe@ccf.org	Cleveland Clinic	Cleveland	Ohio	Nothing to declare

**USA**	Miguel Inca Martinez	incam@ccf.org	Cleveland Clinic Foundation	Cleveland	Ohio	Nothing to declare
**USA**	Anne-Marie Wills	awills@mgh.harvard.edu	Massachusetts General Hospital	Boston	Massachusetts	Nothing to declare
**USA**	Ejaz A. Shamim	ejaz.a.shamim@kp.org	Kaiser Permanente, MidAtlantic Permanente Research Institute	Washington	Washington	Nothing to declare
**USA**	Charles H. Adler	cadler@mayo.edu	Mayo Clinic College of Medicine, Mayo Clinic Arizona	Scottsdale	Arizona	Nothing to declare
**USA**	Ileana Lorenzini	ileana.lorenzini@bannerhealth.com	Banner Sun Health Research Institute	Sun City	Arizona	Nothing to declare
**USA**	Peter Heutink	peter@gp2.org	Global Parkinson's Genetics Program (GP2)	Pacifica	California	Nothing to declare
**Vietnam**	Duan Nguyen		Hue University	Huế		Nothing to declare
**Vietnam**	Toan Nguyen		Hue University	Huế		Nothing to declare
**Vietnam**	NguyễN Thái Thuỳ Ngân	tngan1105@gmail.com	University Medical Center, Ho Chi Minh City	Ho Chi Minh		Nothing to declare
**Vietnam**	Ha Ngoc Le Uyen	uyen.hnl@umc.edu.vn	University Medical Center Ho Chi Minh city	Ho Chi Minh		Nothing to declare
**Vietnam**	Tai Ngoc Tran	tai.tn@umc.edu.vn	University Medical Center HCMC	Ho Chi Minh		Nothing to declare
**Vietnam**	Khang Vo	khang.vnc@umc.edu.vn	University Medical Center	Ho Chi Minh		Nothing to declare
**Vietnam**	Vinh Thanh Nguyen	thanh.nv5@umc.edu.vn	University Medical Center Ho Chi Minh City	Ho Chi Minh		Nothing to declare
**Zambia**	Masharip Atadzhanov	masharip.atadzhanov@gmail.com	University of Zambia	Lusaka		Nothing to declare
